# Multifunctional Calcium–Manganese Nanomodulator Provides Antitumor Treatment and Improved Immunotherapy via Reprogramming of the Tumor Microenvironment

**DOI:** 10.1021/acsnano.3c01215

**Published:** 2023-08-02

**Authors:** Guanghong Luo, Xing Li, Jihui Lin, Gao Ge, Jiangli Fang, Wangze Song, Gary Guishan Xiao, Bo Zhang, Xiaojun Peng, Yanhong Duo, Ben Zhong Tang

**Affiliations:** a School of Medicine, The 2nd Affiliated Hospital, The Chinese University of Hong Kong, Shenzhen (CUHK-Shenzhen), Guangdong 518172, P.R. China; b School of Nursing, Southwest Medical University, Luzhou, Sichuan 646000, China; c School of Medicine, Southern University of Science and Technology, Shenzhen 518055, China; d Department of Neurosurgery, The Shenzhen Luohu Hospital Group, The Third Affiliated Hospital of Shenzhen University, Shenzhen 518001, China; e Department of Radiation Oncology, Shenzhen People’s Hospital (The Second Clinical Medical College, Jinan University; The First Affiliated Hospital, Southern University of Science and Technology), Shenzhen 518020, Guangdong China; f Department of Microbiology, Tumor and Cell Biology (MTC), Karolinska Institutet, Stockholm, 171 77, Sweden; g State Key Laboratory of Fine Chemicals, School of Chemical Engineering, Dalian University of Technology, Dalian, 116024, China; h Research Center for Cancer Metabolism, College of Pharmacology, Shenzhen University of Technology, Chinese Academy of Sciences, Shenzhen, 518118, China; i Key Lab for New Drug Research of TCM, Research Institute of Tsinghua University in Shenzhen, Shenzhen 518057, Guangdong China; j Shenzhen Institute of Aggregate Science and Technology, School of Science and Engineering, The Chinese University of Hong Kong, Shenzhen. Shenzhen 518172, Guangdong China; k Department of Laboratory Medicine, The Third Xiangya Hospital, Central South University, Changsha, 410013, China; l State Key Laboratory of Fine Chemicals, Department of Pharmacology, School of Chemical Engineering, Dalian University of Technology, Dalian, 116024, China

**Keywords:** tumor microenvironment, calcium−manganese nanomodulator, reprogramming, antitumor therapy, immunotherapy

## Abstract

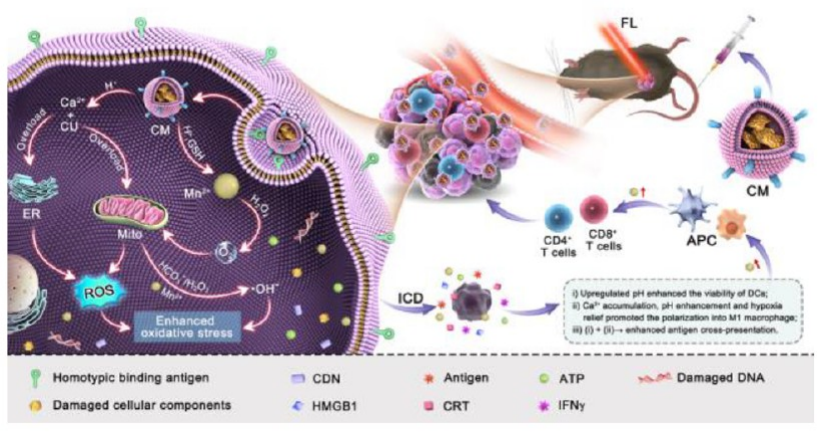

Ions play a vital role in regulating various biological processes, including metabolic and immune homeostasis, which involves tumorigenesis and therapy. Thus, the perturbation of ion homeostasis can induce tumor cell death and evoke immune responses, providing specific antitumor effects. However, antitumor strategies that exploit the effects of multiion perturbation are rare. We herein prepared a pH-responsive nanomodulator by coloading curcumin (CU, a Ca^2+^ enhancer) with CaCO_3_ and MnO_2_ into nanoparticles coated with a cancer cell membrane. This nanoplatform was aimed at reprogramming the tumor microenvironment (TME) and providing an antitumor treatment through ion fluctuation. The obtained nanoplatform, called CM NPs, could neutralize protons by decomposing CaCO_3_ and attenuating cellular acidity, they could generate Ca^2+^ and release CU, elevating Ca^2+^ levels and promoting ROS generation in the mitochondria and endoplasmic reticulum, thus, inducing immunogenic cell death. Mn^2+^ could decompose the endogenous H_2_O_2_ into O_2_ to relieve hypoxia and enhance the sensitivity of cGAS, activating the cGAS-STING signaling pathway. In addition, this strategy allowed the reprogramming of the immune TME, inducing macrophage polarization and dendritic cell maturation via antigen cross-presentation, thereby increasing the immune system’s ability to combat the tumor effectively. Moreover, the as-prepared nanoparticles enhanced the antitumor responses of the αPD1 treatment. This study proposes an effective strategy to combat tumors via the reprogramming of the tumor TME and the alteration of essential ions concentrations. Thus, it shows great potential for future clinical applications as a complementary approach along with other multimodal treatment strategies.

## Introduction

Cancer is a life-threatening disease and is considered as the primary obstacle to the complete development of society.^1^ Many therapeutic modalities, including conventional treatments (surgery, radiotherapy, and chemotherapy) and emerging therapies (immunotherapy, gene therapy, *etc*.) have been developed and explored for their antitumor potential. However, so far, their therapeutic outcomes remain unsatisfactory. Among these available treatments, immunotherapy, which promotes the ability of the body’s immune system to fight against cancer, has several advantages and has emerged as a favorable strategy in the past years. In particular, synthetic monoclonal antibodies that target immune checkpoint proteins have shown great potential in clinical trials.^2^ However, immunotherapy has also some important drawbacks, such as the extremely low response rate^3^ and a series of serious side effects.^4^ Deeper investigations into the molecular mechanisms revealed that the tumor microenvironment (TME) acts as a major contributor to poor treatment responses, which is characterized by a low pH, hypoxia, and high content of reactive oxygen species (ROS) due to the heterogeneity of cellular subpopulations and metabolic activity.^5^ Furthermore, the metabolic status of the cells within tumor tissue, including cancer stem cells (CSCs), stromal cells, cancer-associated fibroblasts (CAFs), and immune cells, allows for reparable points compared to their functions in the cells of normal tissue that benefit the progress of tumor and the therapeutic response. Therefore, the metabolism within the TME has been proposed as an effective therapeutic target for almost all tumors.^6,7^

Under normal physiological conditions, the balance of cellular metabolism is regulated by a complex network. The proper function of key proteins is often dependent on biological ions, especially the metal ions, such as Ca^2+^,^8^ Mn^2+^,^9^ Fe^2+/3+^,^10^ and so on. These play well-established biochemical and nutritional roles for cellular function, growth, and survival, while the induction of an imbalance of ion content usually results in fetal consequences, even death.^11,12^ Moreover, ions and their nanomaterials have been revealed in defending the disease progression in part, especially in antitumor treatment.^12−15^ For instance, calcium ion (Ca^2+^), a kind of essential trace element, has been explored broadly based on its biological roles, including the regulation of intracellular signaling, cellular homeostasis, and immunity of the cell, which thus decides the proliferation, metabolism, and death of various kinds of cells.^16^ Under this circumstance, the oscillation of Ca^2+^ content could be an efficient method to induce various biological processes, in which the alteration of Ca^2+^ content could change the charge properties of phospholipids to promote the activation of T cell receptor and evoke the immune response.^17^ More important, the calcium could serve as a highly effective recruitment agent of macrophages, dendritic cells, and other natural immune cells, to gather them around the disease spot and increase the cells’ ability to phagocytose antigens.^18^ Thus, the excellent therapeutic outcomes of Ca^2+^-based therapeutic application have been achieved.^11,19,20^ For instance, the transformable core–shell nanosonosensitizer (TiO_2_@CaP) can dissolve its CaP shell, releasing Ca^2+^ in an acidic TME and enhancing ROS generation under ultrasound treatment, thereby inducing mitochondrial dysfunction and significantly enhancing immunogenic cell death (ICD), after combination with αPD1 treatment, leading to a systemic and excellent antitumor outcome.^21^ Similar to Ca^2+^, Mn^2+^ has also attracted great attention in tumor treatment due to its roles in the enzymatic transformation of TME. Recently, some studies have reported the Mn^2+^ can prime the cGAS-STING pathway *via* different mechanisms to elicit potent innate and adaptive antitumor immunities for effective tumor suppression effectively.^22,23^ Therefore, Mn^2+^ can significantly improve the therapeutic effects of immunotherapy *via* the reform the TME and the enhancement of immunotherapy.^24,25^ Moreover, the strategies based on two ions, such as the combination of Ca^2+^, Cu^2+^, Mn^2+^, Zn^2+^, Fe^2+^/^3+^, etc., have been proposed and have shown good therapeutic effects in antitumor treatment.^12,15,26,27^ However, the combination of Ca^2+^ and Mn^2+^ dual-ion overloading-mediated tumor therapy has rarely been reported in detail.

**Scheme 1:**
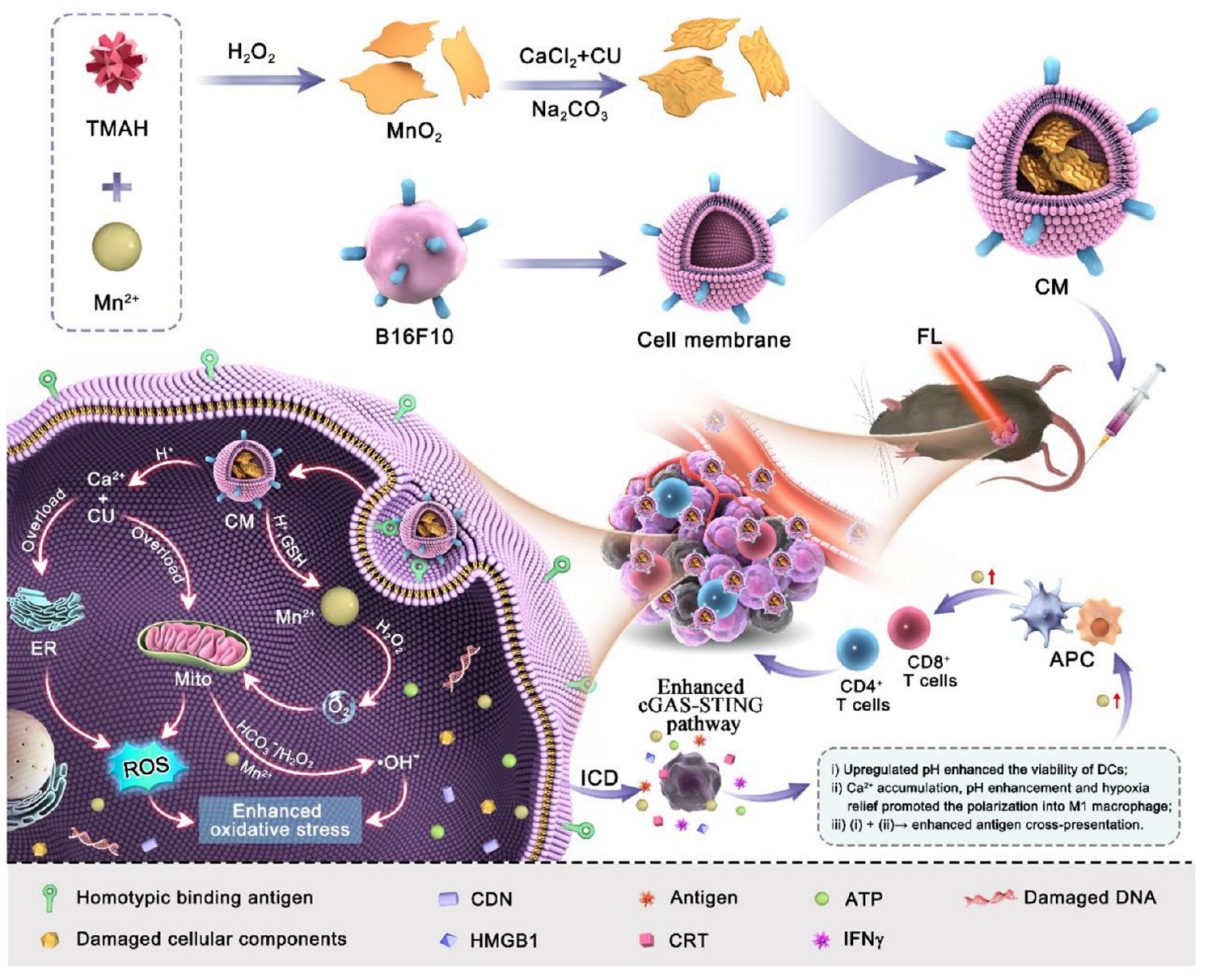
Schematic Diagram of the Multifunctional Calcium–Manganese Nanomodulator Provides Antitumor Treatment and Improved Immunotherapy *via* Reprogramming of the Tumor Microenvironment TMAH: tetramethylammonium hydroxide, CU: Curcumin, ICD: immunogenic cell death, CDN: cyclic dinucleotides, APC: antigen presenting cells, FL: fluorescent imaging.

Inspired by the biological functions of Ca^2+^ and Mn^2+^, we hypothesized that the combination of “Ca^2+^ + Mn^2+^ overloading” could enable effective antitumor treatment with “1 + 1 > 2” synergistic therapeutic effects, and simultaneously providing superior therapeutic effects in combination with immunotherapy. Notably, due to their high metabolic requirements, tumor cells would be more vulnerable to dual ion accumulation than normal tissues,^28,29^ allowing this strategy to have more significant antitumor benefits than other therapeutic modalities. Herein, in this study, we constructed a “dual ion overloading nanomodulator (B16F10@CaCO_3_–CU@MnO_2_, termed CM NPs)” coated with a B16F10 cell membrane. CM NPs could supple surplus Ca^2+^ content (*via* the combination of curcumin [CU], a Ca^2+^ enhancer,^30^ and CaCO_3_) and also increase Mn^2+^ levels (*via* MnO_2_ nanosheets, NSs) in response to the TME by a biomineralization strategy. As illustrated in Scheme 1, the “dual-ion-overloading nanoplatform” exhibited multifunctional characteristics for combating tumors. (i) The B16F10 cell membrane coating guaranteed active tumor targeting, resulting in efficient endocytosis and high accumulation; (ii) The generation of Ca^2+^ and CU was intelligently controlled by a pH-responsive and exhaustion mechanism, enabling Ca^2+^ overload in tumor cells and inducing ICD and subsequent ROS elevations; (iii) The Mn^2+^ further reformed the TME by mitigating hypoxia and enhancing ROS generation and effectively activating the immune system by promoting the proliferation and maturation of immune cells. Given the characteristics of the as-prepared nanoplatform, the dual ion overloading therapy achieved high efficacy against cancer in a synergistic manner. Further, it facilitated an enhanced antitumor response to immunotherapy with an immune checkpoint inhibitor (antibody).

## Results and Discussion

### Synthesis and Characterization of CM NPs

**Figure 1:**
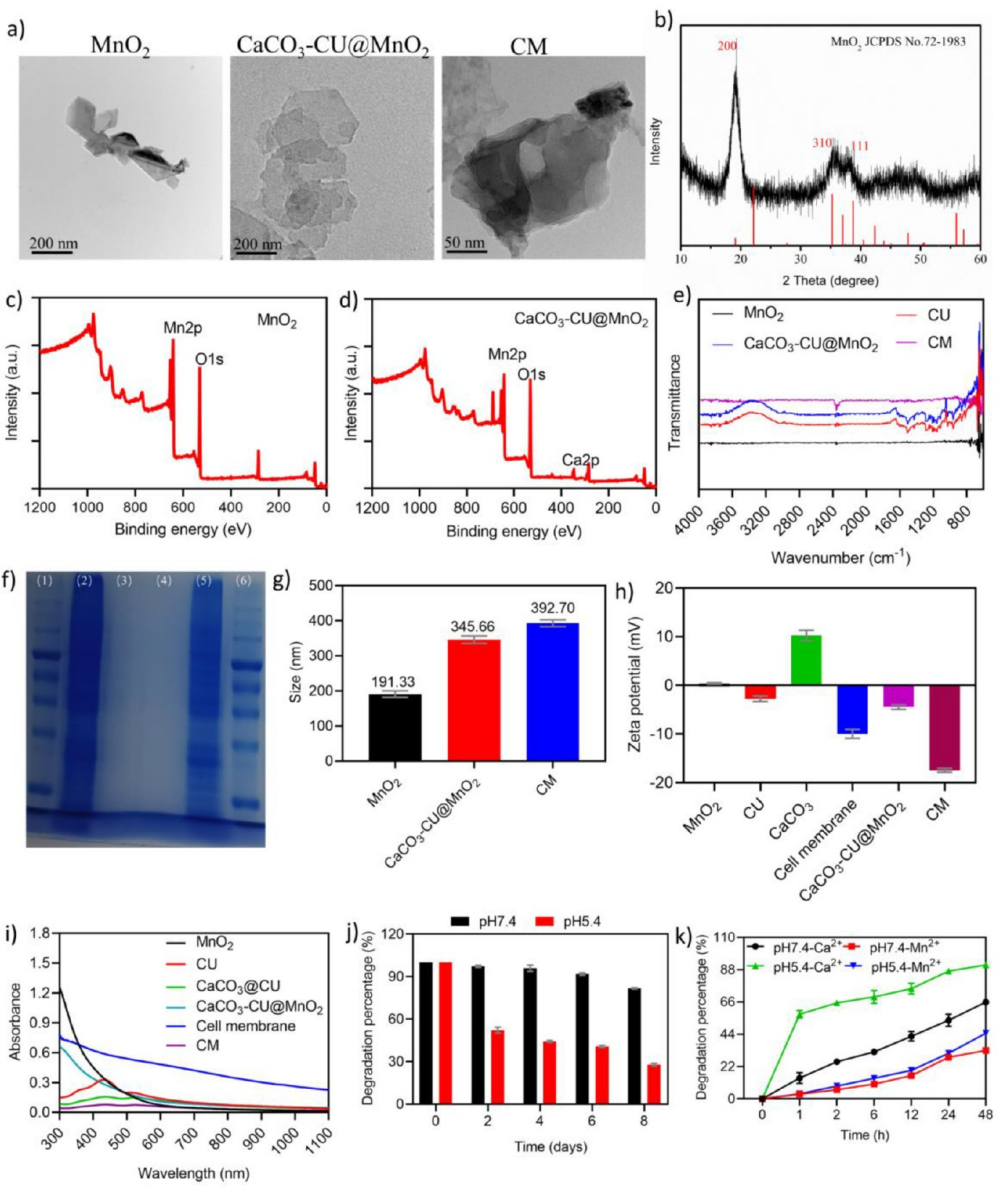
Characterization of CM NPs. (a) TEM of as-prepared MnO_2_ NSs, CaCO_3_–CU@MnO_2_, and CM NPs; (b) XRD analysis of MnO_2_ NSs; (c)-(d) XPS analysis of MnO_2_ NSs and CaCO_3_–CU@MnO_2_ without a cell membrane engulfment; (e) FTIR spectra of various as-prepared materials; (f) SDS-PAGE image, (1) protein marker, (2) B16F10 cell membrane, (3) MnO_2_ NSs, (4) CaCO_3_–CU@MnO_2_, (5) CM NPs, (6) protein marker; (g) DLS analysis of the particle size of MnO_2_ NSs, CaCO_3_–CU@MnO_2_, and CM NPs; (h) DLS analysis of the zeta potential of MnO_2_ NSs, CaCO_3_–CU@MnO_2_, and CM NPs; (i) UV–vis spectra of nanoparticles; (j) quantification of the degradation rate of CM NPs in pH 7.4 and pH 5.4 PBS solutions; (k) quantification of the degradation percentage of Ca and Mn within CM NPs in pH 7.4 and 5.4 PBS buffers. Data are presented as the mean ± SEM.

The preparation of CM NPs is described in Scheme 1, and the detail was shown in the section of materials and methods of the Supporting Information. First, the process of MnO_2_ NSs preparation was referred to a previous study.^31^ TEM results revealed that the as-prepared MnO_2_ exhibited a typical nanosheetlike structure (Figure 1a, left). Then, CaCO_3_–CU@MnO_2_ was prepared by mixing CaCl_2_, CU, and MnO_2_ NSs in a mass ratio of 3:1:3 at 25 °C under magnetic stirring for 90 min. Then, excessive Na_2_CO_3_ was added dropwise to the mixture for 3 h at a high stirring speed. Meanwhile, CaCO_3_@CU was prepared by mixing CaCl_2_ with CU at a mass ratio of 3:1 and adding excessive Na_2_CO_3_ (Figure 1a). Sequentially, CaCO_3_–CU@MnO_2_ was mixed with isolated B16F10 cell plasma membranes for 30 min, and the mixture was physically extruded through a 400 nm polycarbonate membrane 30 times. TEM images showed that the obtained CaCO_3_–CU@MnO_2_ (Figure 1a, middle) and CM NPs (Figure1a, right) had a typical structure.

The as-prepared NPs were examined further. According to energy-dispersive spectroscopy (EDS) analysis, MnO_2_ NSs showed the elemental signals of Mn and O on the surface of MnO_2_ NSs (Figure S1), and these were further confirmed by X-ray diffractometer (XRD, Figure 1b) and X-ray photoelectron spectroscopy (XPS, Figure 1c) analysis. Three typical diffraction peaks of XRD analysis indexed to (200), (310), and (111) corresponding to JCPDS No.72-1983 were observed, along with Mn 2p and O 1s binding energy of XPS analysis at 642.24 and 530.44 eV, respectively; these three analytic results suggested that the MnO_2_ NSs were prepared successfully. The XPS analysis was also applied to systematically examine the as-prepared CM NPs. EDS analysis revealed the elemental signals of Mn, C, O, Ca, N, and S on CM NPs (Figure S2). XPS analysis showed the binding energy of Mn 2p at 642.24 eV, C 1s at 285.31 eV, O 1s at 530.44 eV, and Ca 2p at 347.47 eV for CaCO_3_–CU@MnO_2_ (Figure 1d). Fourier transform infrared spectrometry (FTIR) results in Figure 1e showed strong characteristic peaks at 962, 1154, 1282, 1509, and 1627 cm^–1^ in CU and CaCO_3_–CU@MnO_2_ but not MnO_2_ and CM NPs, and the characteristic peaks were attributed to the vibration of different characteristic functional groups within CU. The results of SDS-PAGE analysis in Figure 1F (lane 2 and 5) suggested the similar protein band in B16F10 cell membrane and CM NPs. The findings above indicated that CM NPs were successfully prepared. DLS analysis demonstrated that MnO_2_, CaCO_3_–CU@MnO_2_, and CM NPs had a hydrodynamic averaged diameter of 191.33 nm (PDI: 0.271), 345.66 nm (0.288), and 392.70 nm (0.297) (Figure 1g and Figure S3) and zeta potentials of +0.38 mV, −4.46 mV, and −17.43 mV, respectively (Figure 1h), where the coloading of CaCO_3_ and CU on the surface of MnO_2_ NSs might be due to the electrostatic adsorption. The UV–vis–NIR spectra of all materials used in this study were also analyzed. MnO_2_ NSs and cell membrane showed no typical absorption peak, the CU (434 nm) CaCO_3_–CU@MnO_2_, and CM NPs showed a typical peak at 434 nm, which the typical peak of CaCO_3_–CU@MnO_2_, and CM NPs was attributed to the existence of CU (Figure 1i). Based on the absorbance value at 434 nm, the content of CU in CaCO_3_–CU@MnO_2_ was calculated to be 38.91%, which was much higher than 7.08% in CaCO_3_@CU at the same feeding ratio. Furthermore, due to the pH-dissociable capability of CaCO_3_, we examined the degradation rate of CM in PBS solutions with different pH values across 8 days. The UV–vis-NIR spectra shown in Figure S4 revealed that CM NPs were degraded in PBS. The degradation rate was quantified based on absorbance at 400 nm and was found to be significantly higher in pH5.4 PBS (72.14%) than in pH7.4 PBS (18.21%) when compared with absorbance on day 0 (Figure 1j), and the morphology of CM NPs suspended in solution with different pH values (5.4 and 7.4) was transformed into NSs with irregular shape and smaller lateral size (Figure S5). Moreover, the release profile of CU from CM NPs also exhibited a pH-dependent manner (Figure S6). Meanwhile, we utilized inductively coupled plasma optical emission spectrometer (ICP-OES) to evaluate the release of Ca^2+^ and Mn^2+^ in the supernatant after centrifugation at predetermined time points. Ca^2+^ levels were rapidly elevated in aqueous solutions with a lower pH value. The degradation rates of Ca^2+^ in pH 5.4 and pH 7.4 solutions and those of Mn^2+^ in pH 5.4 and pH 7.4 solutions containing 50 μg/mL CM NPs were 91.20%, 65.88%, 33.67%, and 32.95%, respectively (Figure 1k), the enhanced release of CU and expose of MnO_2_ nanosheet in acidic solution could benefit from the CO_2_ bubbles.^32^ This revealed the pH-dissociable capability of CaCO_3_ but also indicated that the pH had a marginal effect on MnO_2_ degradation. These results collectively indicated the successful preparation of CM NPs and their low pH-responsive and -dissociable characteristics.

### TME Reprogramming Capacity of CM NPs in Vitro

**Figure 2:**
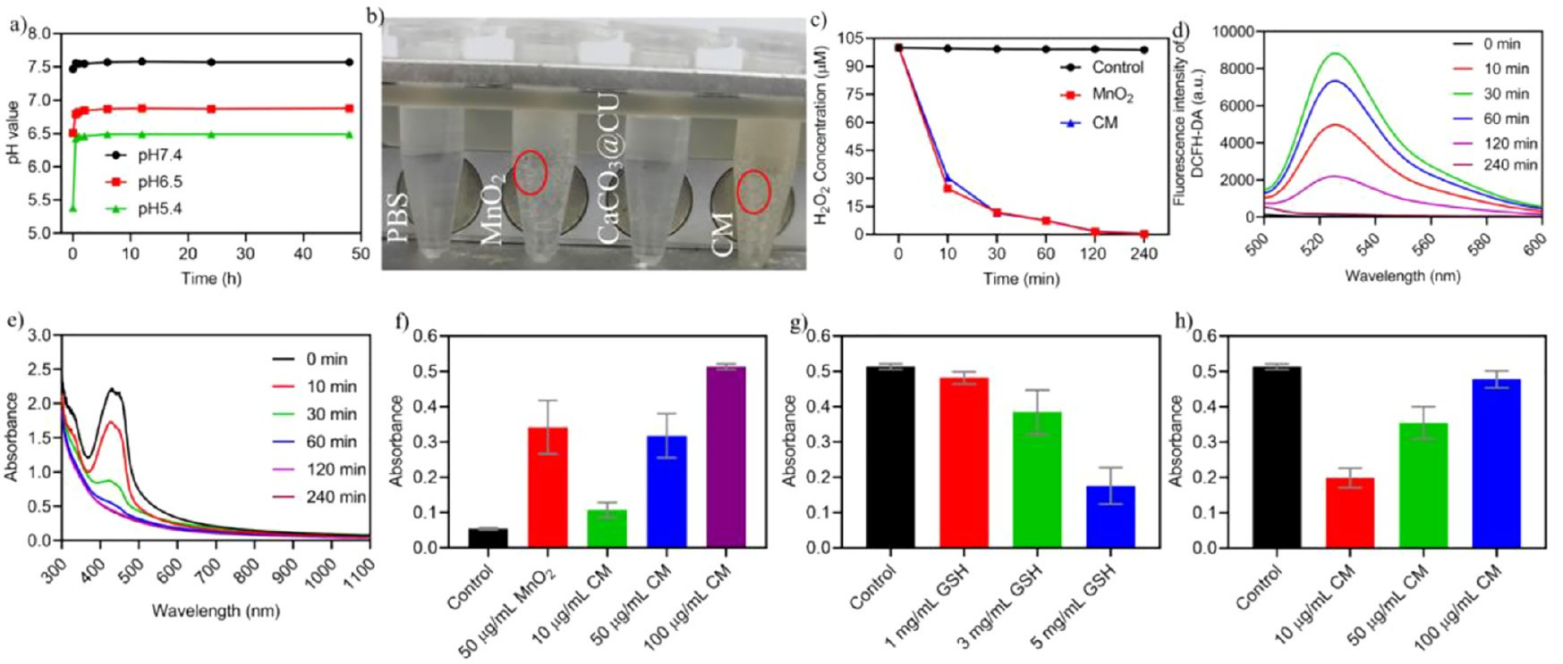
TME reprogramming performance of CM NPs *in vitro*. (a) *In vitro* acidity neutralization profiles of CM NPs; (b) Image of H_2_O_2_ decomposition after treatment with different nanomaterials; red circle, gas bubble; (c) curve of H_2_O_2_ decomposition (*n* = 3); (d) fluorescence spectrum of the ROS probe in the H_2_O_2_ solution containing CM NPs; (e) UV–vis spectrum of DPBF detection in the H_2_O_2_ solution containing CM NPs at different time points; (f) percentage loss of GSH after treatment with different concentrations of CM NPs (*n* = 3); (g) degradation of MB by H_2_O_2_ + CM NPs at different concentrations of GSH (*n* = 3); (h) degradation of MB by H_2_O_2_ + different concentrations of CM NPs in the presence of GSH (*n* = 3). Data are presented as the mean ± SEM.

The TME has been proven to be immunosuppressive and therapeutic resistant that is characterized by a low pH, hypoxia, and high levels of hydrogen peroxide (H_2_O_2_).^33^ For TME, MnO_2_ can act as a nanozyme to relieve hypoxia by decomposing intracellular H_2_O_2_ to remodel the TME.^34^ On the other hand, CaCO_3_ is a biocompatible nanomaterial that can reduce acidity in the TME by reacting with protons and increasing the pH, contributing to ROS generation and the subsequent amplification of intracellular oxidative stress^35,36^ and immune cell polarization and activation.^37^ Thus, MnO_2_ and CaCO_3_ can enhance the therapeutic effects of tumor therapy synergistically. Here, we explore the TME reprogramming capability of CM NPs *in vitro*. The CM NPs were suspended in PBS solutions of different pH values (5.4, 6.5, and 7.4) and stirred at 25 °C. As shown in Figure 2a, the CM NPs could obviously enhance the pH value of the PBS solution *via* the following steps: (i) CaCO_3_ (s) ↔ Ca^2+^ + CO_3_^2–^; (ii) CO_3_^2–^ + 2H^+^ ↔ H_2_CO_3_; and (iii) H_2_CO_3_ ↔ H_2_O + CO_2_. The pH values of 5.4, 6.5, and 7.4 in PBS solution were increased to 6.49, 6.88, and 7.57, respectively, suggesting the excellent acidity-attenuating effect of CM NPs *in vitro*. Meanwhile, there is also CO_2_ generation from CaCO_3_, which exerts no therapeutic effects in cancer treatment.^38^ Then, the decomposition of H_2_O_2_ by CM NPs was measured by adding CM NPs to a H_2_O_2_-containing PBS solution (100 μM). The quantitative analysis of H_2_O_2_ was performed using a Hydrogen Peroxide Assay Kit. The appearance of bubbles (red circle) indicated the decomposition of H_2_O_2_ and the generation of oxygen (O_2_) (Figure 2b), and the quantitative analysis showed that MnO_2_ NSs and CM NPs could decompose H_2_O_2_ quickly *in vitro*. After incubation for 4 h, the H_2_O_2_ was completely decomposed (Figure 2c), and the generation of O_2_ could benefit the relief of hypoxia in the TME.

In the presence of H_2_O_2_, MnO_2_ is known to be a promoter for generating ROS, including ^1/2^O_2_*via* the following catalytic reaction: MnO_2_ + 2H^+^ = Mn^2+^ 2H_2_O + ^1/2^O_2_.^39^ We thus evaluated the generation of ROS in a H_2_O_2_-containing solution in the presence of CM NPs. The ROS probe, DCFH-DA, was mixed into 50 μM H_2_O_2_ at a concentration of 20 μg/mL. The fluorescence intensity was examined using a multifunctional microplate reader (Ex, 488 nm; Em, 525 nm). The fluorescence intensity gradually increased with time until 60 min and then decreased sequentially (Figure 2d), the increase in fluorescence intensity indicated the generation of ROS, while the decrease in fluorescence intensity was attributed to the catalytic decomposition of H_2_O_2_. The ROS content was also measured with 1,3-diphenylisobenzofuran (DPBF), as shown in Figure 2e, as the CM NPs could induce the generation of singlet oxygen (^1^O_2_). ·OH, another toxic ROS, can be generated in the presence of Mn^2+^ ions *via* Fenton-like reactions in physiological NaHCO_3_/CO_2_ conditions.^40^ Due to its capacity to counter intracellular oxidative stress, GSH is upregulated in the cancer cells (0.5–10 mM; normal, 20 μM).^41^ Therefore, we also examined the effect of NPs on GSH inhibition and ·OH generation. The Ellman’s assay (Figure 2f) indicated that CM NPs were effective in inhibiting GSH and reacted with GSH to release Mn^2+^ and GSSG to exhaust cellular GSH that enhances the oxidative stresses. Simultaneously, methylene blue (MB) was used as a ·OH generation probe because it can be degraded in the presence of ·OH. As shown in Figure 2g-h and Figure S7, MB showed significant dose-dependent degradation after incubation with GSH and CM NPs, and the neutral pH buffer enhanced the generation of ·OH. These results suggested that the as-prepared CM NPs would effectively reprogram the TME and induce oxidative stress to execute chemodynamic therapy against cancer cells and benefit other therapeutic modalities.

### Cellular Uptake and Therapeutic Effects in Vitro

**Figure 3:**
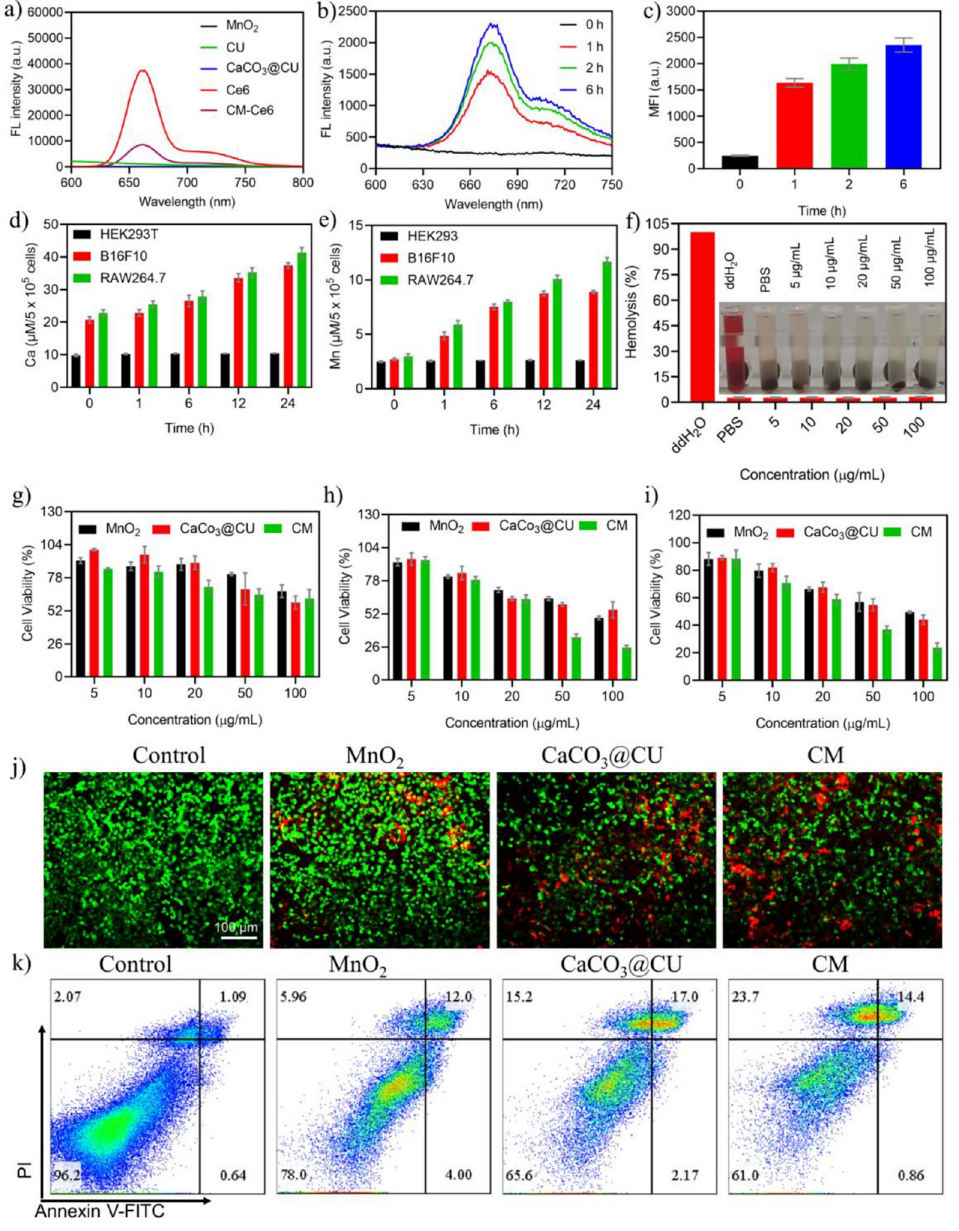
*In vitro* treatment effects of CM NPs. (a) Fluorescence spectrum of Ce6-labeled NPs; (b) fluorescence spectrum of Ce6 in B16F10 cancer cells at different time points; (c) fluorescence intensity analysis of B16F10 cancer cells at different time points; (d)-(e) Manganese and calcium concentration in HEK293T, B16F10, and RAW274.7 cells after incubation for different time points (*n* = 3); (f) hemolysis assays with different concentrations of CM NPs (*n* = 5); (g)-(i) relative viabilities of HEK293T, HeLa, and B16F10 cells after treatment with different types and concentrations of nanomaterials for 24 h (*n* = 4); (j) calcein-AM/PI staining after treatment with different nanomaterials (50 μg/mL); (k) flow cytometry analysis of Annexin V-FITC/PI costained B16F10 cancer cells (50 μg/mL). Data are presented as the mean ± SEM.

To explore the therapeutic effects of CM NPs in cancer cells, we detected the uptake of Ce6-labeled CM NPs (Ce6-CM NPs) in cancer cells. The Ce6 labeled CM NPs was prepared by coloading with CU. As indicated in Figure S8 (UV–vis–NIR spectrum) and Figure 3a (imaging and fluorescence spectrum), the Ce6 was successfully coloaded into CM NPs with a good encapsulation efficacy of 15.71% and retained its NIR fluorescence. As shown in Figure S9 and Figure 3b,c, the CM NPs were endocytosed by B16F10 cells within a brief period, and many NPs were easily internalized by B16F10 cancer cells. Meanwhile, ICP-OES was applied to measure the content of calcium and manganese in B16F10 and RAW264.7 cells after incubation with 50 μg/mL CM NPs for different durations. The analytic results revealed that the CM NPs could be endocytosed by both types of cells, in which the concentrations of calcium and manganese in B16F10 and RAW264.7 cells were 37.38 and 41.29 and 8.90 and 11.68 μM/5 × 10^5^ cells, respectively, and the concentrations increased with the prolongation of the incubation period, but not endocytosed by normal cells significantly (HEK293T) (Figure 3d,e) due to the homing ability of membrane protein.

Subsequently, we examined the therapeutic effects of CM NPs *in vitro* as well as their biocompatibility and biosafety because these are vital prerequisites for clinical translation. A hemocompatibility assay was conducted, and the hemolysis ratio was calculated at different CM NPs concentrations (Figure 3f). Despite an increase in the CM NP concentration, there was no obvious red coloration in the supernatant, suggesting that CM NPs did not cause significant hemolysis. Then, the therapeutic effects of the as-prepared NPs were further tested using the CCK-8 cell counting kit, Calcein-AM/PI costaining assay, and Annexin V-FITC/PI flow cytometry. As shown in Figure 3g-h, a high concentration of CM NPs slightly inhibited cellular viability in normal cells (HEK293T cells). However, severe inhibition was observed in both cancer cell lines (HeLa and B16F10 cells) after incubation for 24 h. This decrease was concentration-dependent that around 60% of treated cells died at 50 μg/mL, suggesting that the increase in Ca^2+^ plus MnO_2_ or Mn^2+^ was effective in tumor cell inhibition. As observed in Figure 3i and 3j, Calcein-AM/PI costaining and flow cytometry assays also indicated that CM NPs had excellent therapeutic effects against B16F10 cells. Importantly, the treatment of MnO_2_, CaCO_3_@CU, and CM NPs could induce the increment of necroptosis.^42^ Meanwhile, the immune cells, such as T cells, are also characterized by abundant content of H_2_O_2_. Here, we also examined the viability of immune cells (RAW264.7 and HT-2). After treatment with different concentrations of CM NPs due to the decomposition of H_2_O_2_, as shown in Figure S10, the CM NPs showed negligible inhibition in immune cells. Overall, the findings demonstrated that CM NPs could kill cancer cells effectively and exhibit good biocompatibility *in vitro*.

### TME Modulation by CM NPs in Cancer Cells

**Figure 4:**
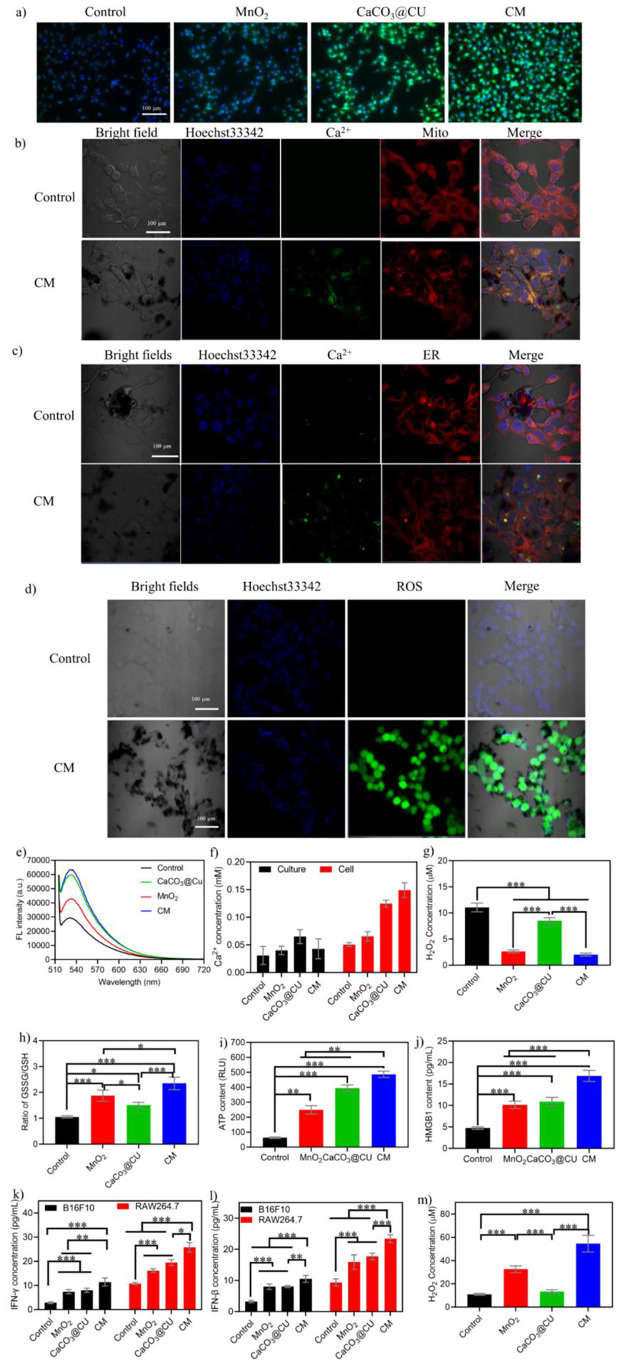
TME modulation and immune activation in cancer cells. (a) Representative images stained by a pH probe after treatment with different nanomaterials; (b) CLSM imaging of Ca^2+^ alteration in mitochondria; (c) CLSM imaging of Ca^2+^ alteration in the ER; (d) representative images showing ROS levels after treatment with CM NPs; (e) fluorescence spectrum of BCECF AM, indicating the changes in intracellular pH values; (f) extracellular (cell culture) and intracellular Ca^2+^ levels (*n* = 3); (g) quantification of intracellular H_2_O_2_ levels after treatment with various nanomaterials (*n* = 3); (h) quantification of intracellular GSSH/GSH after treatment with various nanomaterials (*n* = 3); (i) levels of ATP released after treatment with various nanomaterials (*n* = 3); (j) levels of secreted HMGB1 in cell culture after treatment with various nanomaterials (*n* = 3); (k) IFN-γ levels in RAW264.7 cells after fed with B16F10 cancer cells treated with various nanomaterials (*n* = 3); (l) IFN-β levels in RAW264.7 cells after fed with B16F10 cancer cells treated with various nanomaterials (*n* = 3); (m) H_2_O_2_ levels in M2 macrophages after treatment with various nanomaterials (*n* = 3). Data are presented as the mean ± SEM. *P* values were calculated using one-way ANOVA. *p < 0.05, **p < 0.01, ***p < 0.001, and ****p < 0.0001.

Inspired by the proton neutralization effect of CaCO_3_ and the nanozyme effect of MnO_2_ in the as-prepared CM NPs, as well as their excellent therapeutic effects *in vitro*, we assessed their efficacy of reprogramming the TME in cancer cells, in which the effects of CM NPs in reducing tumor acidity and hypoxia, increasing the tumor levels of Ca^2+^ and ROS, and decreasing the tumor concentrations of H_2_O_2_ and GSH were examined. We measured the alterations in intracellular pH values using the probe BCECF AM due to the acidity of within/outer cancer cells can drive the tumorigenesis and regulate the metabolism, immunity and therapeutic outcomes.^27,43^ The fluorescence intensity of cancer cells was obviously enhanced at 12 h after incubation with MnO_2_, CaCO_3_@CU, and CM NPs compared to the control group (Figure 4a,e, Figure S11). This suggested that both CaCO_3_@CU and CM NPs could efficiently modulate tumor acidity. The alteration of cellular pH can induce significant changes in ion exchange, which further alter the membrane excitability and contractility, the integrity and function of subcellular organelles and interorganelle communication, the generation of toxic free radicals and apoptosis and necrosis.^44^ Then, the mitigation of tumor hypoxia was examined in B16F10 cancer cells after incubation with NPs (50 μg/mL) for different durations with a commercial hypoxia probe (Image-iT Green Hypoxia Reagent). As shown in Figure S12, hypoxia was mitigated significantly after NPs treatment, as indicated by fluorescence imaging and the measured fluorescence intensity. The findings suggested that MnO_2_ and CM NPs are efficient modulators of tumor hypoxia. However, CaCO_3_@CU produced a negligible hypoxia mitigation.

The mitochondria and endoplasmic reticulum (ER) serve as the primary sources for intracellular calcium and primary sites of ROS generation. Given that ROS and calcium are important signaling molecules in biological systems for regulating the hemostasis of oxidative stress^36,45^ and mitochondrial and ER function,^46^ we detected the alterations in Ca^2+^ within cancer cells after incubation with CM NPs (100 μg/mL) using Fluo-4 AM combined with a specific organelle probe. The quantification of the Ca^2+^ concentration was performed using a Calcium Colorimetric Assay Kit. As shown in Figure 4b and 4c, the CM NPs induced an obvious increase in Ca^2+^ signals in the mitochondria and ER, suggesting that treatment with CM NPs could enhance intracellular Ca^2+^ concentrations and disrupt the homeostasis of Ca^2+^, likely resulting in impairment of biological processes. The quantification of Ca^2+^ concentrations in the cells and culture medium revealed that treatment with as-prepared CM NPs (Figure 4f) could alter the content of Ca^2+^ within cells that arose from the synergistic effects of CaCO_3_ and CU (Figure S13).

Cancer cells establish a TME with abnormal metabolism that is conducive to their growth and metastasis, for example, cancer cells show an increased rate of H_2_O_2_ generation, higher than that in normal cells,^47^ which lays the foundation for the generation of highly active ROS.^48^ Hence, we detected the content of H_2_O_2_ and ROS in cancer cells after being treated with CM NPs (50 μg/mL). The results showed that MnO_2_ and CM NPs could induce a significant decrease in H_2_O_2_ (Figure 4g) and GSH (Figure 4 h) concentrations in B16F10 cells, resulting in an obvious increase in ROS levels (Figure 4d). These alterations resulted in enhanced oxidative stresses causing the death of cancer cells. Also, the oxidative damage in mitochondria could induce harm to mitochondrial DNA to generate damaged DNA or cyclic dinucleotides (CDN) and other damaged cellular components, that when released to cytosol induces the cascaded immune response sequentially.^49^

With the neutralization of tumor acidity, mitigation of tumor hypoxia, increase in intratumoral Ca^2+^ and ROS levels, and the decrease in intratumoral H_2_O_2_ and GSH levels, the CM NPs could reestablish the TME. Considering the biological effects of the various components present in CM NPs,^25,50^ we speculated that CM NPs could induce ICD in B16F10 cells arising from the damaged mitochondria and ER, and also the induction of damage-associated molecular patterns (DAMPs).^6,19,51^ More important, the generation of Mn^2+^ could enhance the DNA recognition sensitivity of cGAS to activate and mature the immune system.^52^ Therefore, we evaluated several distinct biomarkers of ICD using Western blotting. As shown in Figure S14, after treatment with the as-prepared NPs, the levels of cGAS, HSP70, HMGB1, CRT, Phos-STING/STING, HMGB1, HSP70, CRT, Phos-TBK1/TBK1, and Phos-IRF3/IRF3 were obviously up-regulated. This suggested that ICD had occurred, and the cGAS-STING pathway had been triggered to initiate immune responses, resulting in DCs proliferation and maturation^29^ and T cell activation.^52^ Encouraged by the results of Western blotting, we examined another ICD marker, ATP, using a commercial kit. As shown in Figure 4i and 4j, CM NPs significantly increased the secretion of ATP and HMGB1 into the cell culture medium, suggesting the activation of cGAS-STING pathway.

The activation of the immune system is initiated by antigen-presenting cells, mainly macrophages and dendritic cells. Hence, RAW264.7 cells were selected to explore the activation of the immune system. We first incubated B16F10 cells with a high concentration of as-prepared NPs (200 μg/mL) to completely kill the cancer cells. The dying cancer cells would be phagocytosed by RAW264.7 macrophages. The components of these cells, including damaged dsDNA, activate the STING signaling pathway and induce the production of IFN-γ and IFN-β. As shown in Figure 4k,l, all the NPs induced the production of IFN-γ and IFN-β that were significantly higher in NP-treated cells than in control cells. Moreover, these levels were higher in cells treated with CM NPs than those in the other two groups. Then, we determined the polarization of the macrophages using different cell markers. As shown in Figure S15, when the induced M2 macrophages were treated with the B16F10 cancer cells after incubation with as-prepared NPs, they could be polarized into M1 macrophages, suggesting that the calcium and manganese could induce the polarization of macrophages to effectively reshape the immunosuppressive microenvironment to favor antitumor immunities (e.g., TAM polarization from M2 to M1).^14,54,55^ And the detection of H_2_O_2_ concentration was also adopted to identify the polarization of M2 into M1 macrophage.^25,56^ As shown in Figure 4m, after the M2 macrophages were treated with the as-prepared NPs (50 μg/mL) for 12 h, a significant increase in H_2_O_2_ levels was found. While CaCO_3_@CU NPs alone showed no significant effect on H_2_O_2_ production, the MnO_2_ NPs and CM NPs showed more obvious effects on H_2_O_2_ production after long-term treatment compared with short-term treatment (1 h).^25^ Moreover, the accumulation of Ca^2+^ could enhance the viability of DCs and improve antigen presentation.^29^ Thus, the synergistic collaboration of DCs and macrophages for antigen cross-presentation could induce the activation of the immune response, recruiting effector T cells to the tumor site to kill cancer cells. Together, these results strongly indicated that the CM NPs could induce ICD and activate the immune system.

### *In Vivo* Targeting, Circulation, and Biodistribution

**Figure 5:**
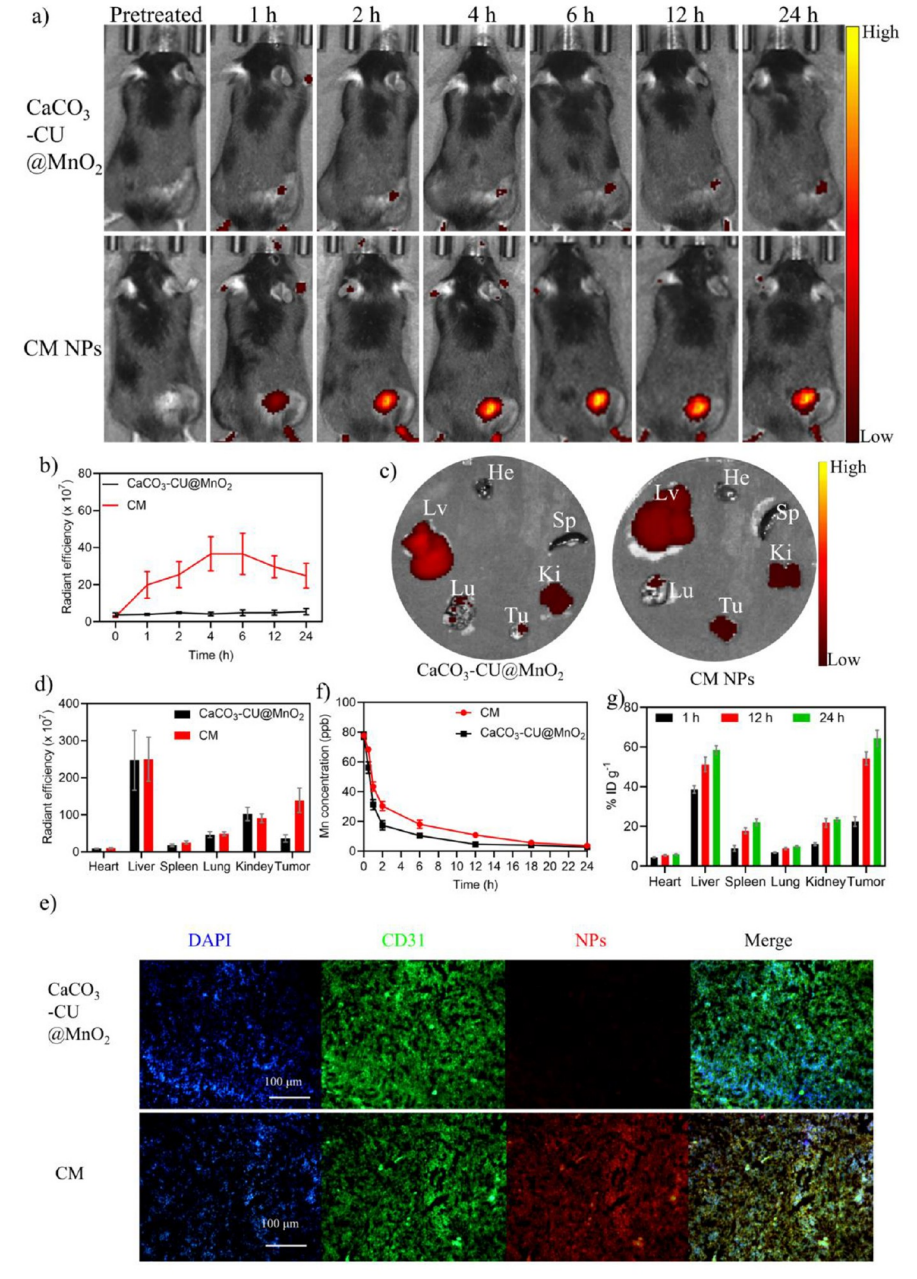
Determination of the targeting, biodistribution, and metabolic mechanism of CM NPs *in vivo*. (a) Representative fluorescence images of NP-treated mice *in vivo* at different time points; (b) radiant efficiency of fluorescence in B16F10 cancer cell-bearing mice at different time points; (c) fluorescence images of major organs and tumors obtained 24 h postinjection. He: heart, Lv: liver, Sp: spleen, Lu: lung, K_i_: kidney, Tu: tumor; (d) radiant efficiency corresponding to (c) (*n* = 3); (e) colocalization of the treated Ce6-labeled CaCO_3_@CU and CM NPs (red), CD31-labeled endothelial cells (green), and nuclei (blue) in tumor sections from B16F10 cell tumor-bearing mice, 24 h after intravenous NP administration; (f) pharmacokinetic profiles of NPs after systemic administration (*n* = 3); (g) biodistribution of CM NPs in B16F10 tumor-bearing mice at different time points (*n* = 3). Data are presented as the mean ± SEM. *P* values were calculated using one-way ANOVA. *p < 0.05, **p < 0.01, ***p < 0.001, and ****p < 0.0001.

Investigations of the systemic biodistribution and clearance of nanotheranostic agents from the body are vital for their potential biomedical applications. Here, we further evaluated whether The CM NPs could accumulate at the tumor sites. We determined their targeting capability based on Ce6 fluorescence using Ce6-labeled CM NPs (Figure S8). The IVIS spectrum *in vivo* fluorescence imaging system was used to examine a B16F10 cancer cell-bearing C57BL/6J mouse model after the intravenous injection of Ce6-labeled CaCO_3_–CU@MnO_2_ or Ce6-labeled CM NPs (15 mg/kg, equal fluorescent intensity), and the in vivo imaging was performed at default time points (0, 1, 2, 4, 6, 12, and 24 h). As shown in Figure 5a, the tumors showed an increased Ce6 fluorescence signal with prolonged time, the fluorescence intensity of which was higher in mice treated with CM NPs than in mice treated with CaCO_3_–CU@MnO_2_, suggesting that coating with B16F10 cell membranes significantly enhanced the targeting capability of the NPs (Figure 5b). After NPs injection for 24 h, the mice were sacrificed, the *ex vivo* fluorescence imaging in the primary organs (heart, liver, spleen, lung, and kidney) and the tumor revealed that the fluorescence intensity was the highest in the tumor tissue, even higher than that in the paired liver tissue (Figure 5c,d), and the fluorescence intensity of isolated tumors in the CM NPs group was also much higher than that in the CaCO_3_–CU@MnO_2_ group. Subsequently, immunofluorescence staining also confirmed that the improved targeting capability of CM NPs benefited from the cancer cell membrane (Figure 5e). We also analyzed the circulating levels of injected NPs and the serum concentrations of Mn^2+^ with ICP-MS as these factors were associated with the amount of NPs reaching the tumor tissue. The circulation and elimination half-lives of CaCO_3_–CU@MnO_2_ and CM in C57BL/6J mice were found to be 1.09 and 15.22 h, 1.63 and 16.19 h, respectively (Figure 5f). The circulation half-life results indicated that cancer cell membrane-coated NPs could decrease the clearance rate from the body. The biodistribution of the CM NPs was detected by measuring the concentrations of Mn^2+^ in the major organs and tumors using ICP-OES at 1, 12, and 24 h after intravenous administration. The analytic results showed that the CM NPs can progressively accumulate at the tumor site, attributed to the targeting capacity provided by the cancer cell membrane and EPR effect (Figure 5g). Collectively, the obtained experimental results suggested that CM NPs showed good tumor targeting capability and pharmacokinetics profiles, with longer blood circulation and a high tumor accumulation efficiency.

### *In Vivo* Anticancer Performance of CM NPs

**Figure 6:**
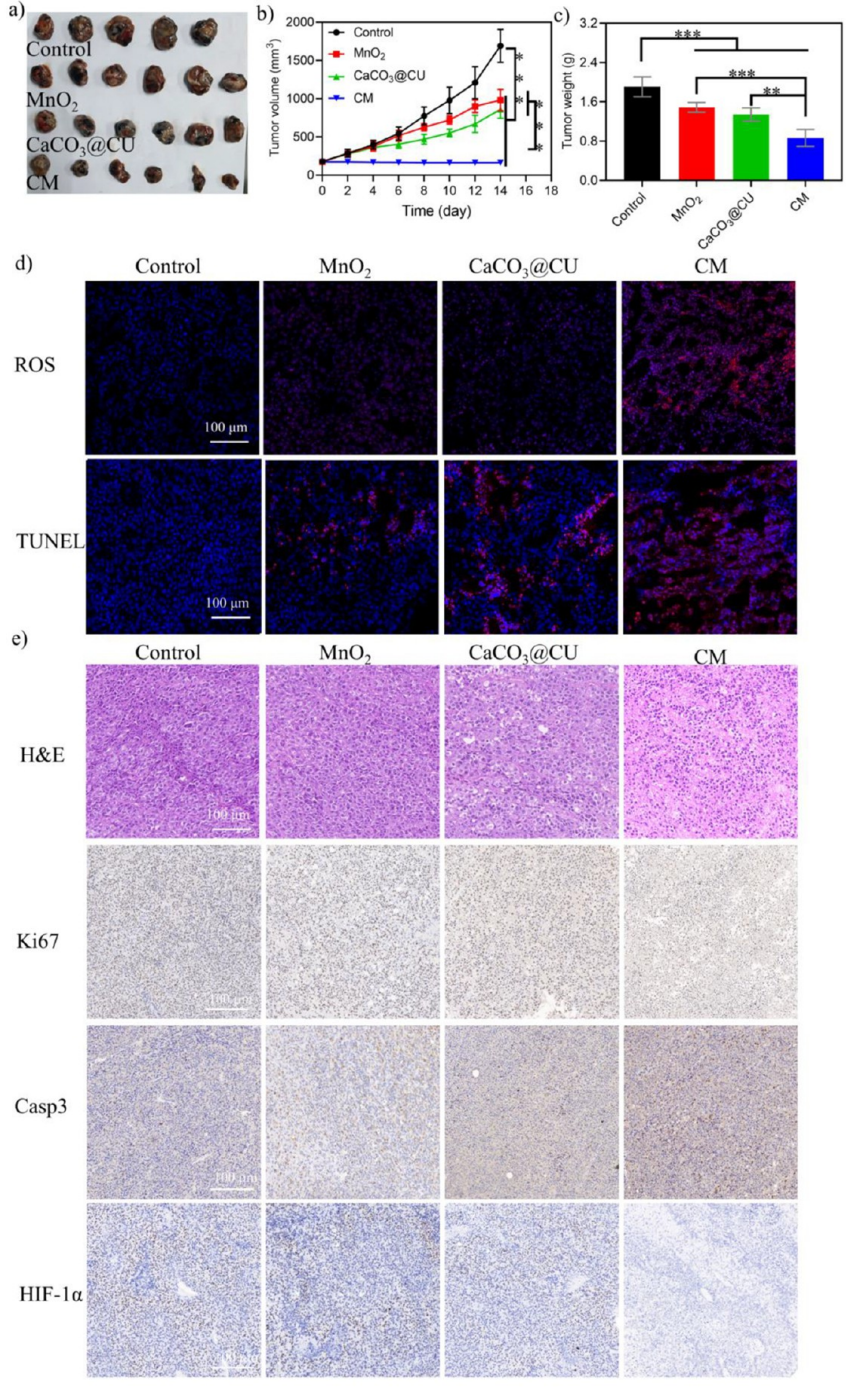
*In vivo* antitumor examination in BALB/c nude mice. (a) Tumors obtained 14 days postinjection; (b) tumor volume growth curves; (c) tumor weight analysis; (d) ROS (red, ROS; blue, DAPI) and apoptosis levels (TUNEL staining) (red, apoptotic cell; blue, DAPI) in tumor tissue; (e) HE staining and IHC staining of apoptotic proteins in tumor sections after different treatments. Data are presented as the mean ± SEM. *P* values were calculated using ordinary one-way ANOVA. *p < 0.05, **p < 0.01, ***p < 0.001, and ****p < 0.0001.

CM NPs were effective in reprogramming the TME and antitumor *in vitro* and also showed a good targeting capability and accumulation at tumor sites. Thus, we carefully tested their potency in killing cancer cells *in vivo*. B16F10 tumor-bearing BALB/c nude mice were randomized to one of four groups when the tumor volume reached ∼180 mm^3^ (*n* = 6 per group): (1) control; (2) MnO_2_; (3) CaCO_3_@CU; and (4) CM (15 mg/kg). Mice in group (1), the control group, were treated with an equivalent volume of PBS, and the mice in groups (2)-(4) received intravenous injections of the corresponding NPs on day 0. The continuous therapeutic effectiveness in terms of tumor size, body weight, and survival was recorded every 2 days. The MnO_2_ and CaCO_3_@CU groups showed a certain extent of tumor inhibition when compared with the control group (Figure 6a,b). However, CM NPs exhibited the best antitumor effects. The tumor growth inhibition (TGI) rate in the control, MnO_2_, CaCO_3_@CU, and CM groups was −655.40%, −469.79%, −385.09%, and +6.15%, respectively. When the treatment period ended, mice were sacrificed, and tumors were obtained (Figure 6a,c). These analyses confirmed the good antitumor efficacy of CM NPs and the other two NPs. Moreover, it is possible that the CM NPs and MnO_2_ and CaCO_3_@CU NPs failed to eliminate tumors or provide stronger tumor regression more significantly because the dose of injected NPs was lower than that in previous studies.^35^ Here, we also examined ROS levels and apoptosis rates in frozen tumor sections. As shown in Figure 6d, an obvious increase in ROS generation and apoptosis was observed after treatment with MnO_2_, CaCO_3_@CU, and CM NPs compared with the control group, and the most obvious increase in fluorescence signals was observed in the CM group. The H&E staining and IHC staining of these sections after different treatments suggested that the arrangement of cells was the loosest in group (4), and showed significantly less nuclear staining and greater nuclear fragmentation, suggesting that CM NPs induced extensive cancer cell death and their effect was greater than that of MnO_2_ and CaCO_3_@CU. IHC staining revealed that tumors from groups (2), (3), and (4) had a significantly altered expression of apoptotic proteins. For example, Ki67 was downregulated, and Caspase-3 was upregulated. Moreover, the IHC staining of HIF-1α (hypoxic indicator) suggested the relief of hypoxia *in vivo* (Figure 6e). The aforementioned findings were indicative of the good antitumor effects of CM NPs.

On the other hand, it is important to examine treatment toxicity and biosafety. During the treatment period, the body weights of mice in all four groups remained largely constant (Figure S16a) and there was a natural death of the mouse (Figure S16b). Hence, MnO_2_, CaCO_3_@CU, and CM NPs did not produce obvious systemic toxicity. Meanwhile, systematic biosafety investigations, including routine blood examinations, blood chemistry, and HE staining of main organs, were also performed. As shown in Figure S17, the blood biochemistry indicators were comparable among the four groups. HE staining revealed no evident destruction or lesions in the primary organs of mice from all four groups (Figure S18). We concluded that the pH-responsive CM NP system enabled Ca^2+^ content overload and the generation of Mn^2+^ to reprogram the TME, and these effects were critical for tumor regression. Moreover, the system showed good biosafety.

### *In Vivo* Immunotherapeutic Anticancer Performance of CM NPs

Because the increase of ROS and O_2_ in tumor tissue could polarize the M2 macrophage into M1 macrophage^57^ and due the capability of calcium/manganese to reshape the immunosuppressive microenvironment to favor antitumor immunities,^18,55^ we previously also confirmed the *in vivo* antitumor effects of CM NPs and validated their ICD induction capacity. This contributes to the activation of the immune system and turns a “cold” tumor into a “hot” tumor, leading to sustained antitumor effects. Therefore, we investigated the effectiveness of this strategy in inducing antitumor immune responses in a C57BL/6J mouse model of B16F10 tumors. The mice were divided into the (1) Control; (2) MnO_2_; (3) CaCO_3_@CU; and (4) CM groups. The body weights in all groups were comparable after 14 days of treatment, indicating the tolerable safety profiles of all as-prepared nanomaterials and dosages (Figure S19). This was further confirmed with the examination of routine blood parameters (Figure S20a), blood chemistry indicators (Figure S20b), and H&E staining of the primary organs (Figure S21).

**Figure 7:**
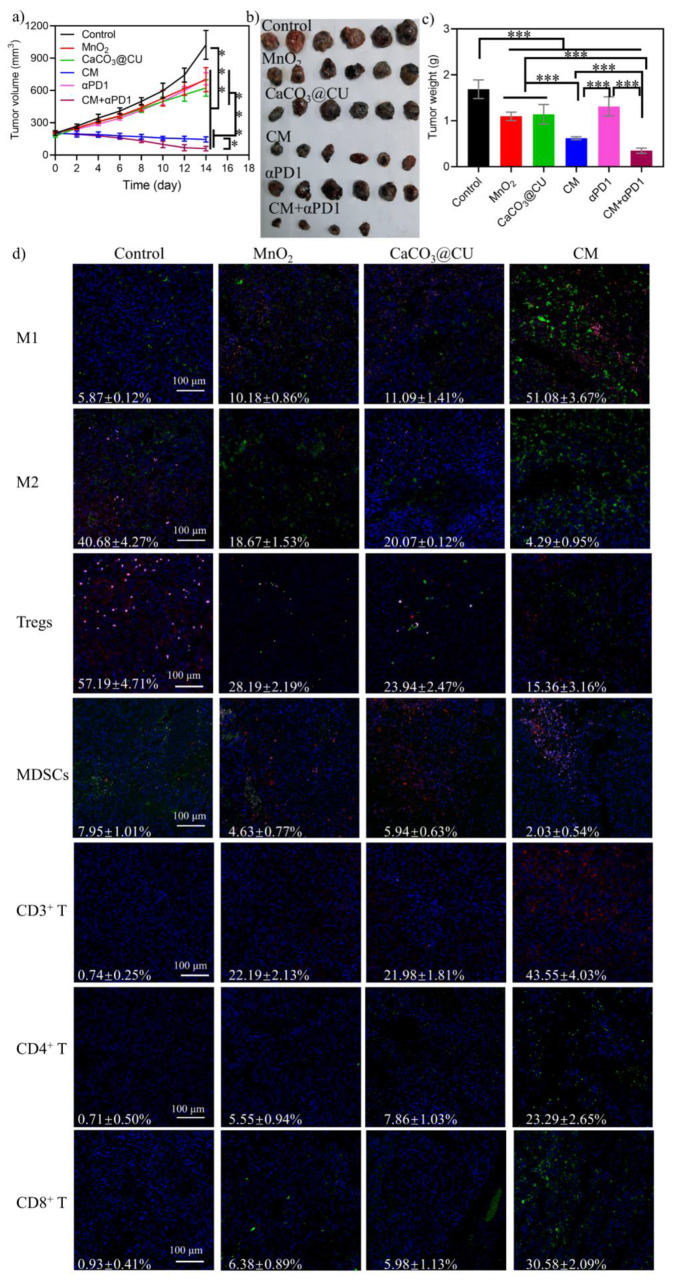
*In vivo* antitumor immunotherapy study in B16F10 cancer cells bearing C57BL/6J mice. (a) Images of tumors obtained on day 14 postinjection; (b) tumor volume growth curves; (c) tumor weight analysis; (d) fluorescence imaging of various immune cell subpopulations, i.e., M1 macrophages: CD11b^+^(red)/F4/80^+^(green)/CD80^+^(pink), M2 macrophages: CD11b^+^(red)/F4/80^+^(green)/CD206^+^(pink), Tregs: CD3^+^(red)/CD4^+^(green)/Foxp3^+^(pink), MDSCs: CD45^+^(red)/CD11b^+^(pink)/Gr-1^+^(green), total T cells: CD3^+^(green), CD4 T cells: CD4^+^(green), cell nucleus, were stained with DAPI (blue). Data are presented as mean ± SEM. *P* values were calculated using one-way ANOVA. *p < 0.05, **p < 0.01, ***p < 0.001, and ****p < 0.0001.

For tumor inhibition assessments, the same aforementioned treatments used in BALB/c nude mice were applied, and the tumor size and survival were also recorded every 2 days. When the treatment period ended, mice were sacrificed and tumors were collected and weighed. The MnO_2_ and CaCO_3_@CU NPs alone could partially suppress tumor regression compared with the control group (Figure 7a and b). We found that treatment with the CM NPs could significantly suppress the growth of these B16F10 tumors (Figure 7a and b). The TGI rates observed after treatment with PBS, MnO_2_, CaCO_3_@CU, and CM NPs in B16F10 tumor-bearing mice were −403.89%, −203.78%, −238.15%, and +20.09%, respectively. Additionally, the measurement of tumor weights also confirmed that CM NPs provided stronger inhibitory effects than did the other three groups (Figure 7c). Furthermore, evaluations for the detection of ROS and apoptosis showed that mice treated with MnO_2_, CaCO_3_@CU, and CM NPs exhibited an increase in the level of generation of ROS and apoptosis in tumor tissues (Figure S22). Moreover, the H&E staining (Figure S21) and IHC staining for Ki67, Bcl-2, and Caspase-3 (Figure S23) also revealed evident cellular death after NPs injection compared with the control group. More precisely, we carefully investigated the antitumor immunity in C57BL/6J mouse models of B16F10 tumors. The tumor tissues were isolated from the aforementioned groups, the whole protein of tumor tissue was extracted for the evaluation of ICD, and various types of immune cells were identified and examined using fluorescence staining based on canonical markers of immune cell subpopulations based on their function.^22,58^ Similar to the results *in vitro*, the results of Western blotting also suggested that the CM NPs could induce the generation of ICD significantly (Figure S24) and induced the increased generation of cytokines including IFN-γ and IFN-β (Figure S25). The results of fluorescence staining of immune cells from tumor tissue slices are shown in Figure 7d. The overload of Ca^2+^ combined with Mn^2+^ enhanced the intratumoral abundance of M1 macrophages. Meanwhile, it remarkably decreased the intratumoral abundance of M2 macrophages, Tregs, and MDSCs, which are all immunosuppressive types of immune cells. This suggested that the CM NPs activated antitumor immunity by inducing macrophage polarization from the M2 into the M1 phenotype. Moreover, the examination of staining for total T cells, effector T cells, and cytotoxic T cells also revealed that these cell populations were significantly elevated in the treatment groups compared to the control group. Moreover, the effects of CM NPs were stronger than those of MnO_2_ and CaCO_3_@CU alone (Figure 7d). On the other hand, the results in Figure 7d show that the treatment groups exhibited obvious effector T cell infiltration compared with the control group, which could be attributed to the activation of the cGAS-STING signaling pathway by Mn^2+^. The results confirmed that treatment with CM NPs can elicit a strong antitumor immunity.

Simultaneously, we also explored the antitumor responses induced by αPD1 combined with CM NPs *in vivo* based on the activation of immune responses. Twelve B16F10 cancer cell-bearing C57BL/6J mice were randomized into two groups. One group was treated with αPD1 (group 5) and the other with CM and αPD1 (group 6). The antitumor effects were then examined in the C57BL/6J mice. The dose of αPD1 was 100 μg/mouse; moreover, this antibody was administered *via* intraperitoneal administration on day 1, 4, and 8. As shown in Figure 7a-c, the treatment of mice with αPD1 could slightly suppress their tumor growth. This could be due to the poor targeting capability of αPD1 *in vivo*. When combined with CM NPs, αPD1 produced synergistic therapeutic effects (Figure 7a-c), and two tumors in the CM + αPD1 group disappeared. The antitumor effects were also examined using ROS and apoptosis examination, H&E staining, and IHC staining (Figure 7c and Figures S22, S23). Moreover, to verify the therapeutic effects of as-prepared cancer cell membrane capsulated NPs, we also prepared PANC-1 cancer cell membrane-coated CM NPs for combating pancreatic cancer cells, as shown in Figures S26 and S27. The CM NPs exhibited excellent antitumor outcomes combined with ICB and also exhibited good biosafety. Collectively, these results suggested that the CM NPs could enhance the antitumor responses induced by αPD1 and provide synergistic therapeutic effects.

## Conclusion

In summary, we prepared a nanomedicine platform consisting of CM NPs that was capable of exhibiting pH-responsive drug release and reprogramming the TME *via* several steps in response to the components of the TME (Scheme 1). The CM NPs enabled Ca^2+^ overloading, evoking ICD, and reversed the immunosuppressive state of the TME. Because CaCO_3_ can effectively react with protons, the CM NPs could act as efficient proton scavengers and deplete intracellular protons, reversing tumor acidity and increasing the pH value. This enabled increased ROS generation. The Mn^2+^ generated from MnO_2_ NSs consumed H_2_O_2_ and induced the generation of ROS, while the Ca^2+^ from CaCO_3_ and CU could destroy the calcium pool in the mitochondria and ER. This resulted in ICD and activated the immune response, which was in turn attributed to the ICD and the biological effects of Mn^2+^ on the immune system. After tail vein injection, the CM NPs showed enhanced accumulation at tumor sites, with good biosafety, and exhibited antitumor effects and antitumor immunity against B16F10 tumors. Moreover, the CM NPs enhanced the antitumor response produced by αPD1. However, it is important to examine the antitumor therapeutic effects with a higher dose of as-prepared NPs. In summary, the findings from this study provide an effective strategy for reprogramming the TME by altering the content of essential ions in the body and achieving antitumor effects in the body. Further, they show the great potential of this strategy as a complementary approach to enhance the effects of other therapeutics, especially in immunotherapy of cancers, and provide multimodal clinical treatment in the future.

## Methods

### Preparation of MnO_2_ NPs

The preparation of MnO_2_ NSs was conducted following a previous report.^31^ In brief, 18 mL of 0.5 mM tetramethylammonium hydroxide (TMAH) was added into 10 mL of 0.2 mM MnCl_2_ tetrahydrate solution and mixed well, and then 2 mL of hydrogen peroxide was dropwise added rapidly into the mixture above to form a black-brown suspension. The suspension was then stirred for 24 h at room temperature (rt) without light irradiation. The MnO_2_ NSs was collected with centrifugation at 13,000 rpm for 10 min and washed with ethanol and ddH_2_O thrice. Lastly, the MnO_2_ NSs were dispersed in ddH_2_O at 4 °C for future experiment.

### Preparation of CaCO_3_–CU@MnO_2_

The 3 mg of as-prepared MnO_2_ was gently resuspended into 10 mL of ddH_2_O containing 3 mg of CaCl_2_ and 1 mg of CU under moderate stirring for 6 h, and then the overdose of Na_2_CO_3_ (10 mL, 500 μg/mL) was added into the mixture rapidly and kept under high stirring speed in the dark overnight. Finally, the CaCO_3_–CU@MnO_2_ was collected with centrifugation at 13,000 rpm for 10 min and washed with ethanol and ddH_2_O thrice and resuspended in ddH_2_O for further experiments. The loading efficiency of CU was estimated using the absorbance intensity from the UV–vis spectrum, and the concentration of CU was calculated with a standard curve (C0 and C1(supernatant)), and the loading capability (%) was estimated: (C0–C1)/C0 × 100. The same method was applied to obtain CU@CaCO_3_. Meantime, the chlorin e6 (Ce6) was also mixed with CaCl_2_ and CU to obtain fluorescent labeled nanoparticles.

## Supporting Information Available

Analytical techniques, synthesis, and characterization of CM NPs, in vitro treatment of CM NPs against cancer cells, *in vivo* tests of CM NPs with tumor mouse model, and blood parameters as well as hematoxylin- and eosine staining (PDF)
