# Supplementary material for: Multifunctional Calcium–Manganese Nanomodulator Provides Antitumor Treatment and Improved Immunotherapy via Reprogramming of the Tumor Microenvironment
Source: ACS Nano. 2023 Aug 2;17(16):15449–65. doi: 10.1021/acsnano.3c01215 (PMC10448754; doi:10.1021/acsnano.3c01215)
Supplement: Supplementary file 1 — nn3c01215_si_001.pdf [file nn3c01215_si_001.pdf]

Supplementary Material

Multifunctional Calcium-Manganese Nanomodulator Provides Antitumor Treatment and Improved Immunotherapy via Reprogramming of the Tumor Microenvironment

Guanghong Luo[1,5,†], Xing Li[3,†], Jihui Lin[1,2,†], Gao Ge[11], Jiangli Fang[6], Wangze Song[12], Gary Guishan Xiao[8,12], Bo Zhang[1,4,*], Xiaojun Peng[7], Yanhong Duo[5,6,9,*], Ben Zhong Tang[10]

[1]School of Medicine, The 2nd Affiliated Hospital, The Chinese University of Hong Kong, Shenzhen (CUHK-Shenzhen),Guangdong, 518172, P.R. China

[2]School of Nursing, Southwest Medical University, Luzhou, Sichuan, 646000, China.

[3]School of Medicine, Southern University of Science and Technology, Shenzhen 518055, China.

[4]Department of Neurosurgery, The Shenzhen Luohu Hospital Group, The Third Affiliated Hospital of Shenzhen University, Shenzhen 518001, China.

[5]Department of Radiation Oncology, Shenzhen People's Hospital (The Second Clinical Medical College, Jinan University; The First Affiliated Hospital, Southern University of Science and Technology), Shenzhen 518020, Guangdong, China.

[6]Department of Microbiology, Tumor and Cell Biology (MTC), Karolinska Institutet, Stockholm, Sweden.

[7]State Key Laboratory of Fine Chemicals, School of Chemical Engineering, Dalian University of Technology, Dalian, China.

[8]Research Center for Cancer Metabolism, College of Pharmacology, Shenzhen University of Technology, Chinese Academy of Sciences, Shenzhen, China.

[9]Key Lab for New Drug Research of TCM, Research Institute of Tsinghua University in Shenzhen, Shenzhen 518057, Guangdong, China.

[10]Shenzhen Institute of Aggregate Science and Technology, School of Science and Engineering, The Chinese University of Hong Kong, Shenzhen. Shenzhen 518172, Guangdong, China.

[11]Department of Laboratory Medicine, The Third Xiangya Hospital, Central South University, Changsha, China.

[12]State Key Laboratory of Fine Chemicals, Department of Pharmacology, School of Chemical Engineering, Dalian University of Technology, Dalian, China.

[†]These authors contributed equally to this work.

*Corresponding author.

Experimental Section

Materials

Manganese dioxide (MnO_2) NSs was synthesized by referencing to previous study[1] and stored in ddH_2O at 4 °C and used for the further experiment. Manganese chloride ($MnCl_2$), Calcium chloride ($CaCl_2$), sodium carbonate (Na_2CO_3) and tetramethylammonium hydroxide were purchased from Macklin Inc. Curcumin (CU) was obtained from MCE. αPD1 antibody was purchased from Biolegend. Cell Counting Kit-8 (CCK-8), Reactive Oxygen Species Assay Kit, Fluo-4 AM, Calcium Colorimetric Assay Kit, Annexin V-FITC/PI Apoptosis Detection Kit, Hydrogen Peroxide Assay Kit, ATP Assay Kit, BCECF AM, Mito-Tracker Red CMXRos and ER-Tracker Red were bought from Beyotime Biotechnology. Calcein-AM and PI were purchased from YEASEN Biotechnology Co., Ltd. PBS (pH 7.4) and fetal bovine serum (FBS) was purchased from Gibco Life Technologies (AG, Switzerland). DMEM, trypsin-EDTA and penicillin-streptomycin were purchased from Corning. The information of antibodies was described in the section of western blotting and fluorescent staining. All other chemicals used in this study were analytical reagent grade and used without further purification. Ultrapure water (18.25 MΩ.cm, 25 °C) was used to prepare all the solutions.

Preparation of $CaCO_3$-CU@MnO_2

The 3 mg of as-prepared MnO_2 were gently re-suspended into 10 mL of ddH_2O containing 3 mg of $CaCl_2$ and 1 mg of CU under moderate stirring for 6 h, and then the over-dose of Na_2CO_3 (10 mL, 500 μg/mL) were added into the mixture rapidly and kept under high stirring speed in the dark overnight. Finally, the $CaCO_3$-CU@MnO_2 was collected with centrifugation at 13,000 rpm for 10 min and washed with ethanol and ddH_2O thrice and resuspended in ddH_2O for further experiments. The loading efficiency of CU was estimated using the absorbance intensity from the UV-Vis spectrum, and the concentration of CU was calculated with standard curve (C0 and C1(supernatant)), the loading capability was estimated:(C0-C1)/C0 x 100%. The same method was applied to obtain CU@$CaCO_3$. Meantime, the chlorin e6 (Ce6) was also mixed with $CaCl_2$ and CU to obtain fluorescent labelled nanoparticles.

Preparation of nanoplatform (CM NPs)

Firstly, B16F10 cancer cells were cultured in DMEM medium replenished with 10% fetal bovine serum and penicillin-streptomycin. To collect cellular membrane, the cells were fully collected with 2 mM EDTA in pre-cold PBS. After washing with pre-cold PBS thrice by centrifugation, the cells were suspended and disrupted in a 10 mL hypotonic lysing buffer (10 mM Tris-HCl (pH 7.5), 10 mM KCl, 2 mM $MgCl_2$ and protease inhibitor), and homogenized using a Dounce homogenizer. The homogenized solution was centrifuged at 3500 g for 5 min at 4 °C to collect the supernatant. The supernatant was then centrifuged at 20,000 g for 15 min, after which the pellets were discarded and the supernatant was centrifuged again at 100,000 g for 30 min. The plasma membrane pellets were then washed once in 10 mM Tris-HCl buffer, and resuspended in PBS and stored at -80 °C for further experiments. The protein content of obtained cellular membrane was quantified using a BCA kit (Beyotime).

To prepare cancer cell membrane vesicles, the membrane material (200 μg) and $CaCO_3$-CU@MnO_2 (5 mg) was physically extruded through a 450 nm polycarbonate membrane (Millipore, USA) for 30 passes. The obtained CM NPs was centrifuged at 12,000 g for 15 min to pellet the cell membrane coated $CaCO_3$-CU@MnO_2 rather than free vesicles. The same method was applied to obtain B@MnO_2 (coined as MnO_2) and B@$CaCO_3$-CU ($CaCO_3$@CU).

Characterization

Scanning Electron Microscope (SEM) images were taken on the ZEISS SUPRA® 55 Scanning electron microscope. Transmission Electron Microscope (TEM) images were taken on the JEM-3200FS Transmission Electron Microscope (Japen). X-ray diffractometer (XRD) was conducted on the D8 Advance (Bruker, Germany, Voltage ≤ 40 kV, $10^{\circ} \leq 2$ Theta $\leq 80^{\circ}$). Fourier Transform Infrared Spectrometer (FTIR) were conducted on the Frontier FTIR Spectrometer (PerkinElmer, USA, 600 cm^{-1} ≤ wavelength ≤ 4000 cm^{-1}). ICP-OES results were acquired from an inductively coupled plasma mass spectrometer (JY2000-2, Horiba Jobinyvon, USA). UV-Vis-NIR absorption spectra were recorded on a SPARK 10M (TECAN, USA) or NanoDrop One^{C} Microvolume UV-Vis Spectrophotometers. The zeta potential, size and polydispersity index (PDI) of nanoparticles were measured on ZETASIZER (Nano

Series, Malvern). The intensity of fluorescence was evaluated on a SPARK 10M (TECAN, USA) with fluorescent cuvette. The fluorescence images were obtained from a laser scanning confocal microscopy (TCS SP8, Leica, Germany). *In vivo* biodistribution was detected using a small animal imaging system (IVIS Spectrum, PerkinElmer, USA).

SDS PAGE protein analysis

Membrane proteins were analyzed *via* SDS-PAGE. Briefly, B16f10 cell membrane, MnO_2 and $CaCO_3$@CU without cell membrane coating, together CM NPs samples were suspended in 1 x SDS sample buffer at concentrations determined based on a BCA assay kit. Samples were then boiled to 100 °C for 10 min, after which 20 μg per sample was separated *via* 10% SDS-PAGE for 1.5 h at 120 V, with the resultant gels being stained using Coomassie Blue for 2 h, washed overnight with ddH_2O, and imaged.

In vitro Ca^{2+} *and* Mn^{2+} *release*: CM NPs (100 μg/mL) was sufficiently dissolved into PBS buffer (pH 7.4 and pH 5.4) for different time points, respectively. Subsequently, the mixture was centrifuged at 13,000 rpm for 15 min, the supernatant was collected for detecting Ca^{2+} and Mn^{2+} concentrations by ICP-OES.

Stability Performance of NPs

The 100 μg/mL (Mn ion concentration) of CM NPs were dispersed in PBS solution with different pH value (pH 5.4 and pH7.4) at rt for 8 days. The change of CM NPs stability was evaluated using imaging and UV-Vis spectrum.

Quenching of H_2O_2 by CM NPs

For the quenching experiment, CM NPs or as-prepared MnO_2 NSs (50 μM Mn) was suspended in PBS, H_2O_2 (100 μM) was added to initiate the reaction. For *in vitro* reactivity of as-prepared NPs toward H_2O_2, the concentration of H_2O_2 was measured using Hydrogen Peroxide Assay Kit. For *in vitro* cellular reactivity of as-prepared NPs toward H_2O_2, RAW264.7 cells were pretreated with 25 ng/mL IL-4 to generate M2 macrophages, then incubated with 20 μg/mL of as-prepared NPs for 12 h. The amount of intracellular H_2O_2 was quantified using Hydrogen Peroxide Assay Kit.

Acidic Attenuation Effect of NPs *in vitro*

The as-prepared CM NPs (20 μg/mL) was suspended in PBS with different pH

values (pH 5.4, 6.5 and 7.4), stirring at rt and evaluating the pH value using a pH meter at default timepoints (0, 0.5, 1, 2, 6, 12, 24 and 48 h).

Detection of GSH Depletion

Ellman's assay was employed to measure the depletion of GSH content by the as-prepared NPs. Briefly, 200 μL of different concentrations of NPs were mixed with 200 μL of 1 mM GSH solution and co-incubated under shaking with a speed of 180 rpm for 3 h. then, the NPs were separated and removed by centrifuging at 12,000 rpm for 10 min. Then 700 μL of 0.05 M Tris-HCl (pH= 8.0) solution and 10 μL of 5,5'-Dithiobis-(2-nitrobenzoic acid) (DTNB,100 mM in DMSO) were added into the supernatant. The absorbance of the mixture at 410 nm was measured by UV−Vis absorption spectroscopy.

Generation of •OH by the CM NPs

First, the generation of •OH by a Mn^{2+}-catalyzed Fenton-like reaction was evaluated. The 25 mM $NaHCO_3$/5% CO_2 buffer or PBS buffer solutions contained 10 μg/mL of MB, 20 mM of H_2O_2, and 20 μg/mL CM NPs were incubated at 37 °C for 60 min. The generation of •OH was revealed by increasing absorption of MB at 665 nm. Next, the •OH generation ability of the CM NPs was investigated. The NPs was incubated with different concentrations (0, 1, 3 and 5 mg/mL) of GSH solution ($NaHCO_3$/5% CO_2 buffer) for 60 min. After centrifugation at 12,000 rpm for 10 min, the mixture of MB (10 μg/mL) and H_2O_2 (20 mM) in $NaHCO_3$/5% CO_2 buffer were added to the supernatant and incubated at 37 °C for 0.5 h in a shaker before recording the UV−Vis spectra. The CM NPs solution with the equivalent concentration was used as the control group.

Cell culture

The 293T, Hela and RAW 264.7 cells were stored in our lab, B16F10 and PANC-1 cells were obtained from the Cell Bank of the Shanghai Fuheng Biotechnology Co., Ltd. (Shanghai, China). 293T, HeLa, RAW 264.7, B16F10 and PANC-1 cells were cultured in Dulbecco's Modified Eagle's Medium (DMEM, Gibco) media with 1% penicillin-streptomycin and 10% fetal bovine serum (Gibco) at 37 °C in 5% CO_2.

Cellular toxicity assay

293T, HeLa and B16F10 cells were cultured on a 96-well plate (2 x 10^4 cells/well), the cells were incubated in a humid chamber of 5 % CO_2 and 95% humidity at 37 °C. After the confluence of cells in 96-well plate up to 60-65%, the culture medium was replaced with 200 μL of new full culture medium that contained the desired amount of CM NPs (0, 5 ,10, 20, 50 and 100 μg/mL). Six multiples were set for every sample and the cells were treated with samples for specific interval for 24 h. then 10 μL CCK-8 and 90 μL serum free culture medium mixed gently and added into each well, incubated for 1 h 37 °C. The cell viability was evaluated by measuring the absorbance at the wavelength of 450 nm of SPARK 10M. The following formula was used to calculate the toxicity of cells: Cell viability (%) = (mean of Abs. value of treatment group/mean Abs. value of control) ×100%, which the control group were the blank group.

Assessment of cellular uptake efficiency

HEK293T, B16F10 and RAW264.7 cells were seeded at 35 mm confocal dish at 1×10^5 per dish and cultured overnight. After that, cells were washed with PBS for thrice and incubated with CM NPs (labeled with Ce6) for 6 h. After incubation, cells were collected by trypsin digestion and fluorescence intensity was detected by a flow cytometer (BD DxFLEX, American). And the cells were washed by PBS thrice, collected and measured the concentration of manganese and calcium by ICP-OES.

Phagocytosis *in vitro*

First, B16F10 cells were cultured overnight into six-well plates with 2 × 10^5 cells/well seeding density. When the confluency was up to 70%, cells were treated with the following groups: MnO_2 (10 μg/mL), $CaCO_3$@CU (10 μg/mL), CM (10 μg/mL) and Control (PBS). After these treatments, the culture supernatant of B16F10 cells was collected for IFNγ detection. Meanwhile, these treated B16F10 cells were placed under the UV radiation (1500 J cm^{-2}) to kill all the cells. Then, the dying B16F10 cells were collected and fed to RAW264.7 cells. After the phagocytosis of dying B16F10 cells, the culture supernatant of RAW 264.7 cells were collected and prepared for IFNγ detection.

Hemolysis assay

The blood samples were collected from C57BL/6J mice. In general, 1 mL blood sample was diluted with 5 mL of PBS, and then red blood cells (RBCs) were separated from the serum by centrifugation (2000 rpm,10 min). After washing with PBS six times,

the RBCs were diluted with 10 mL of PBS. Then the RBCs were treated with different concentrations of *CM NPs* solution (5, 10, 20, 50 and 100 μg/mL), and the ddH_2O and PBS were set as positive and negative controls to evaluate hemolysis ability. After incubated and placed in the table concentrator at 37 °C at a speed of 180 rpm for 3 h, the mixtures were centrifuged at 10,000 rpm for 10 min. Finally, the absorbances of supernatants at 570 nm were recorded by a microplate reader to calculate the percent hemolysis.

***In vitro* antitumor efficiency**

To determine the antitumor efficiency *in vitro* toxicity of the nanoplatform, B16F10 cells were seeded in a 6-well plate and cultured with various NPs for 24 h followed examined by CCK-8 Assay, Live and Dead Cell Staining and Flow Cytometry Detection under the manual instruction of CCK-8 Assay Kit, Calcein-AM/PI and Annexin V-FITC Kit, respectively.

Detection of ATP levels

B16F10 cells were seeded in 24-well plate at a density of 5×10^4 cells per well for 16 h (the confluence up to 80-85%), then the cell culture was replaced with fresh culture medium that containing various different concentration of NPs (50 μg/mL) and cultured for another 24 h, then the cells were collected and evaluated culture ATP content with ATP assay kit under the instruction of the provider's manual. The bioluminescence was detected and recorded using SPARK 10M plate reader.

Evaluation of ROS production

B16F10 cells were incubated with various concentration of CM NPs (50 μg/mL) for 12 h, then, culture medium was replaced with fresh serum-free medium containing 5 μM of DCFH-DA for a further incubation of 30 min without light. Then, CLSM were applied for ROS analysis.

Intracellular Ca^{2+} concentration assay

The intracellular Ca^{2+} concentration of B16F10 cells was detected by the fluorescent dye Fluo-4 AM and Calcium Colorimetric Assay Kit (Beyotime, China). B16F10 cells were seeded on 12-well plates with a density of 5×10^4 cells/well for 18-20 h, then treated with various NPs. After incubation for 12 h, the cells were stained with 3 μM Fluo-4 AM in serum free DMEM culture for 30 min at 37 °C, then washed thrice with PBS. The fluorescence intensity was detected at an excitation wavelength

of 488 nm and emission wavelength of 526 nm by CLSM. For quantifying the concentration, the cells were collected and lyzed with lysate buffer. Next, 75 μL Chromogenic Reagent and 75 μL Calcium Assay Buffer were added to the 50 μL lysates, followed by the 5 min incubation, the absorbance value at 575 nm were recorded using microplate reader.

Measurement of intracellular pH (pHi)

The intracellular pH was measured using BCECF-AM in a SPARK 10M Multi-Mode Microplate Reader. Briefly, 1×10^4 B16F10 cells were grown overnight in 48-well plates. After the cells incubated with MnO_2, $CaCO_3$@CU and CM NPs for 6 h at 37 °C in 5 % humidified CO_2, the cell culture was removed and washed thrice with serum-free DMEM, then, cells were incubated in serum-free DMEM containing 1 μM BCECF-AM for 15 min at 37 °C in 5 % humidified CO_2. The cells were then washed with PBS thrice and incubated in PBS. The intensity of BCECF-labeled cells was recorded the fluorescence emission intensity at 530 under the excitation at 440 and 490 nm. And the relative pHi were presented to the intensity of fluorescence.

Western blotting

Whole cell protein lysates were boiled after diluted using 5 × SDS loading buffer and 30 μg of total protein was subjected to different densities (8-15%) of sodium dodecyl sulfate (SDS), then transferred onto a polyvinylidene fluoride membrane. The membranes were blocked with blocking buffer (Beyotime Biotechnology, cat. no. P0023B) for 1 h and incubated with the following primary antibodies overnight at 4 ˚C: cGAS (cat.no: ab224144, Abcam), STING (cat.no.13647S, CST), Phos-STING (cat.no: AP1369, Abclonal), Calreticulin (CRT, cat.no: ab92516, Abcam), HMGB1 (cat.no: ab79823, Abcam), TBK1 (cat.no: A3458, Abclonal), Phos-TBK1 (cat.no: AP1026, Abclonal), IRF3 (cat.no: A11373, Abclonal), Phos-IRF4 (cat.no: A19717, Abclonal)and β-actin (cat. no. AC026, Abclonal), β-actin was set as loading control. The membranes were washed thrice for 5 min in a mixture of PBS and Tween-20 (PBST), incubated with anti-rabbit IgG, HRP-linked second antibody secondary antibodies (cat. no. 7074; CST) for 2 h, and washed again thrice for 5 min in PBST. Immunoblotting bands were observed using ChemiScope Series 6000Touch(Clinx Science Instruments Co.,Ltd) after incubated with Pierce™ ECL Plus western blotting substrate (cat. no. 32132; Life).

Immunofluorescent staining

Cells or tissue slices samples were fixed with 4% paraformaldehyde for 15 min at rt. Next, the samples were permeabilized with 0.1% Triton X-100 PBS for 10 min and cultured with a blocking buffer containing 5% bovine serum albumin (BSA) for 1 hour at rt. Then, the samples were incubated overnight with specific primary antibodies at various dilution at 4 °C overnight, washed with PBS three times, and incubated in specific secondary antibody for 2 h at rt. Last, the sample were stained with DAPI, washed with PBS, and imaged using an Leica CLSM (SP8). For the determination of immune cell subpopulation, the specific antibody of canonical marker were listed in the section of results detailedly.

Animals and tumor models

Female BALB/c nude mice and C57BL/6J (5-6 weeks old) were purchased from GemPharmatech Co.,Ltd, All animals were housed in a specific pathogen-free (SPF) laboratory in the Animal Center of Shenzhen People's Hospital at 22 ± 1 °C temperature and 40-50% humidity under a 12 h light/dark cycle with free access to water and standard laboratory chow. All procedures were approved by the Institutional Ethics Committee for Animal Experimentation and were conducted in accordance with the Shenzhen People's Hospital. B16F10 cells bearing tumor models were established by subcutaneously injecting B16F10 cancer cells (1×10^5 cells suspended in 100 μL of PBS) or PANC-1 cancer cells (1×10^7 cells suspended in 100 μL of PBS) into the flank of each mouse. The tumors allowed to grow to ~150 mm^3 for further use. In the *in vivo* tumor inhibition experiment, mice were divided randomly into 4 groups, and each group contained 6 mice.

***In vivo* evaluation of tumor targetability and distribution**

When the B16F10 tumor volume bearing in C57BL/6J mice reached ∼150 mm^3, the mice were treated with the intravenous injection of Ce6 labelled CM NPs (100 μL). Next, the mice were placed in IVIS imaging systems for the observation of the fluorescence images at default time (0 and 24 h). 24 h later, the mice were anesthetized, and the tumors and major organs (liver, heart, lung, spleen and kidney) were harvested to analyze the fluorescence distribution. The main organs, including heart, liver, spleen, lung, kindey and tumor were collected to measure the concentration of Mn element at

day 0, 12 and 24 h. At same time, 30 μL blood was collected from tail vein and determined the concentration of Mn element to evaluate the blood circulation and metabolism of NPs.

***In vivo* tumor inhibition**

B16F10 tumor bearing in BALB/c nude mice were randomly divided into four groups (Each group contained 6 mice): PBS (control), MnO_2, $CaCO_3$@CU and CM. B16F10 tumor bearing C57BL/6J mice were randomly divided into four groups (Each group contained 6 mice): PBS (control), MnO_2, $CaCO_3$@CU, CM, αPD1 and CM + αPD1. The mice were intravenously injected with 100 μL of PBS, or as-prepared NPs (15 mg kg^{-1}) for per mouse at days 0, 3 and 7, where the mice group of αPD1 and CM + αPD1 were treated with intraperitoneal administration of αPD1 antibody (100 μg in 100 μl saline per mouse) on days 1, 4, and 8. respectively. Mouse weight and tumor size were measured every 2 days. The tumor volume was calculated as length × $(\text{width})^2$ × 1/2. The tumor growth inhibition (TGI) rate was calculated following equation:

$TGI = (V_C - V_T)/V_C \times 100\%$

where V_T is the tumor volume after the treatments, and V_C is the tumor volume of the control group.

Two weeks later, the mice were sacrificed, and the main organs (heart, liver, spleen, lung, and kidney) and the tumors were collected for HE staining analysis, and the blood were also collected for blood routine and biochemical analysis. The routine blood measurement included white blood cell (WBC) counts, RBC counts, hemoglobin (HGB), mean corpuscular volume (MCV), mean corpuscular hemoglobin (MCH), mean corpuscular hemoglobin concentration (MCHC), platelets (PLT), and hematocrit (HCT). The blood chemistry tests included alanine transaminase (ALT), aspartate transaminase (AST), total protein (TP), globulin (GLB), total bilirubin (TBIL), blood urea nitrogen (BUN), creatinine (CREA), and albumin (ALB).

TUNEL Assay

Tumor tissue slices was treated using a TUNEL assay kit. In this experiment, apoptotic cells were stained with Red and were analyzed through a fluorescence microscope (Eclipse 80i, Nikon Corporation).

Statistical analysis

The experimental results were expressed as means ± the standard error of mean (SEM). The significance of difference was analyzed by one-way analysis of variance followed by the Tukey's honestly significant difference (HSD) test using Gpaphpad prime 8.0 (GraphPad Software) as $^{*}p < 0.05$, $^{**}p < 0.01$, $^{***}p < 0.001$ and $^{****}p < 0.0001$.

Supplemental figures

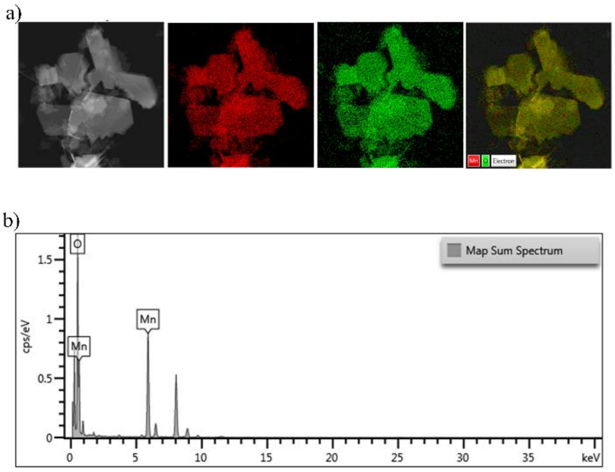

Figure S1. STEM-EDS analysis of MnO_2 NSs. (a) EDS elemental mapping of MnO_2 NSs; (b) Distribution of Mn and O element in MnO_2 NSs.

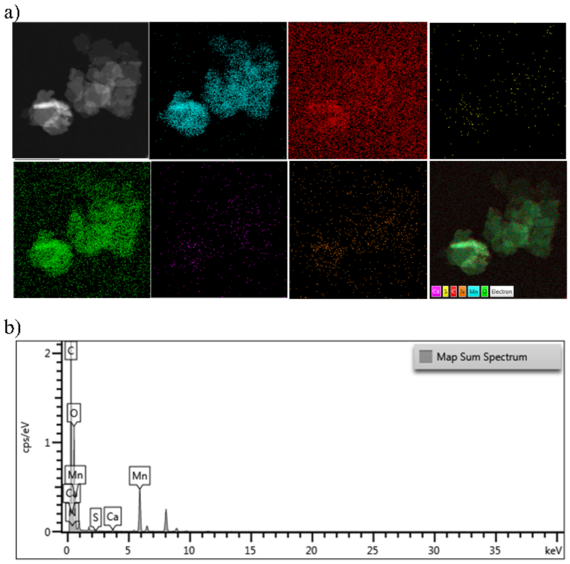

Figure S2. STEM-EDS analysis of CM NPs. (a) EDS elemental mapping of CM NPs; (b) Distribution of Mn, Ca, S, N, C and O element in CM NPs.

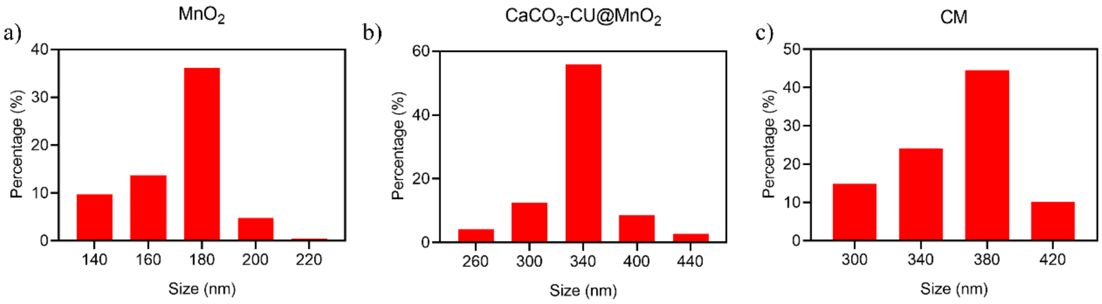

Figure S3. DLS analysis of size of MnO_2 (a), $CaCO_3$-CU@MnO_2 (b) and CM NPs (c).

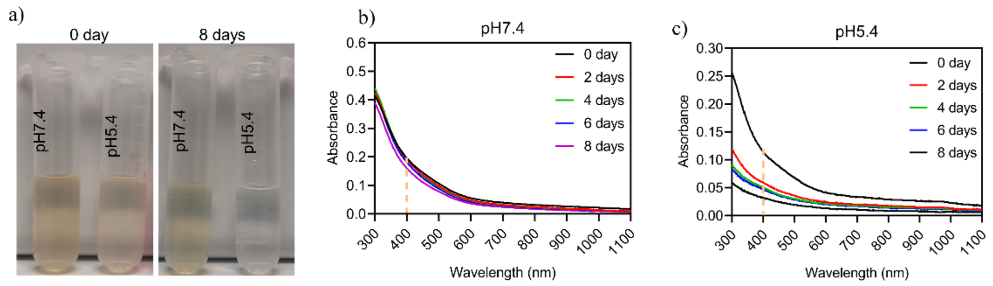

Figure S4. Degradation behaviors of CM NPs *in vitro*. (a) Images of CM NPs in PBS solutions of different pH values at different timepoints; (b) UV-Vis spectrum of CM NPs at pH 7.4 PBS solution; (c) UV-Vis spectrum of CM NPs in a pH5.4 PBS solution;

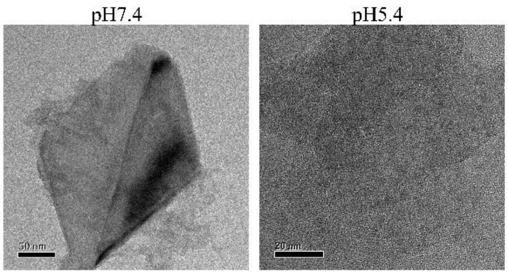

Figure S5. TEM of CM NPs suspended in solution with different pH value for 24 h.

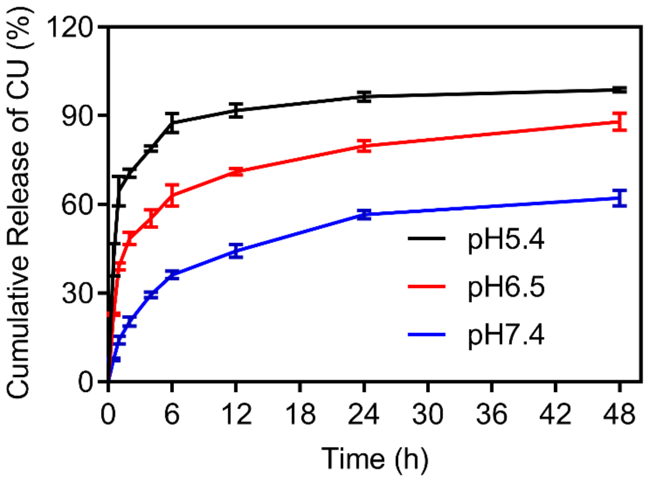

Figure S6. The cumulative release of CU in solution with different pH values.

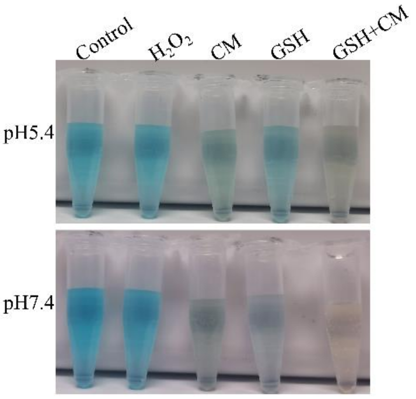

Figure S7. Images of MB degradation in H_2O_2 contained various pH value solution after treated with CM NPs.

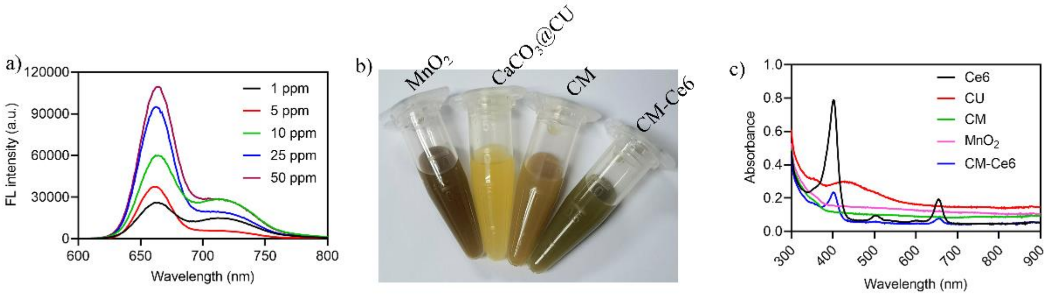

Figure S8. The characterization of Ce6 labelled NPs. (a) Fluorescent spectrum of different concentration of Ce6; (b) Images of different NPs; (c) UV-Vis spectrum of Ce6 labelled NPs.

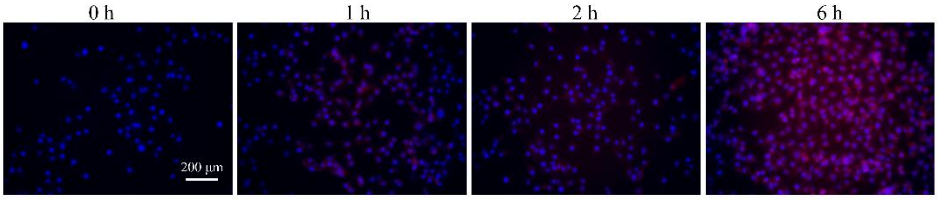

Figure S9. Fluorescent images of B16F10 cell uptake after incubated with Ce6 labelled CM NPs.

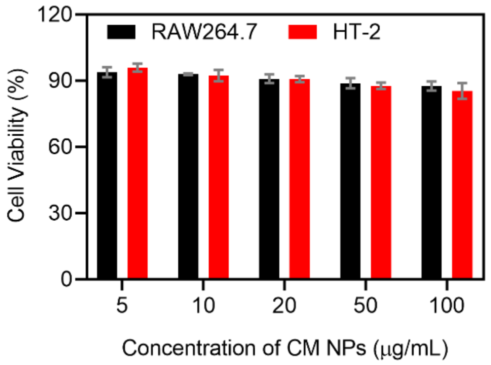

Figure S10. Relative viabilities of RAW264.7 and HT-2 cells after incubated with different concentration of CM NPs.

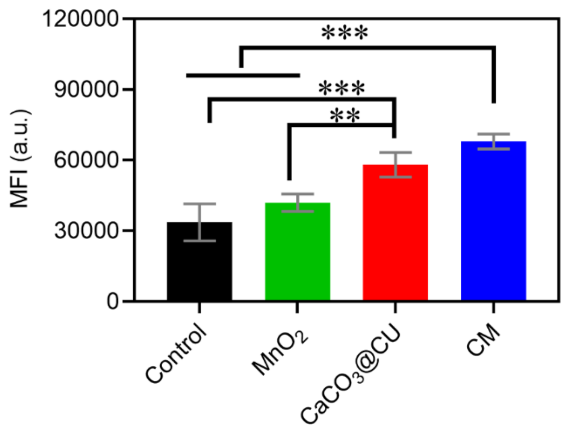

Figure S11. Quantification of fluorescent intensity of BCECF AM (n=3).

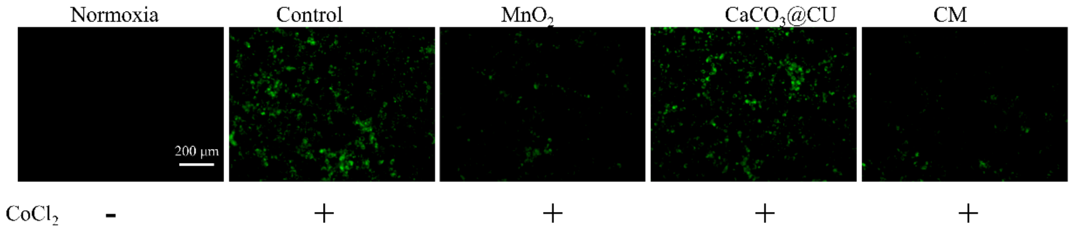

Figure S12. Representative images of hypoxia in $CoCl_2$ contained B16F10 cancer cells after treated with various nanomaterials.

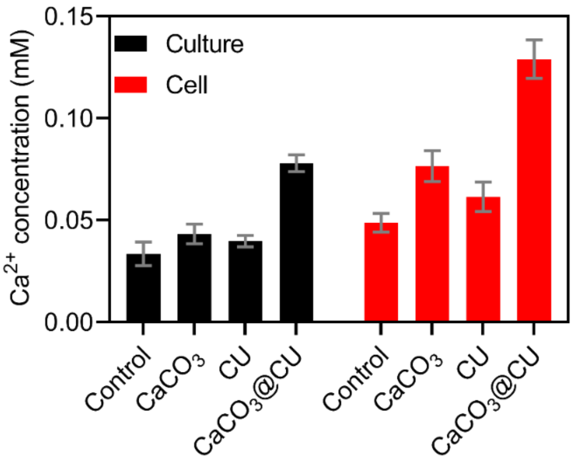

Figure S13. Quantification of Ca^{2+} concentration after different treatment.

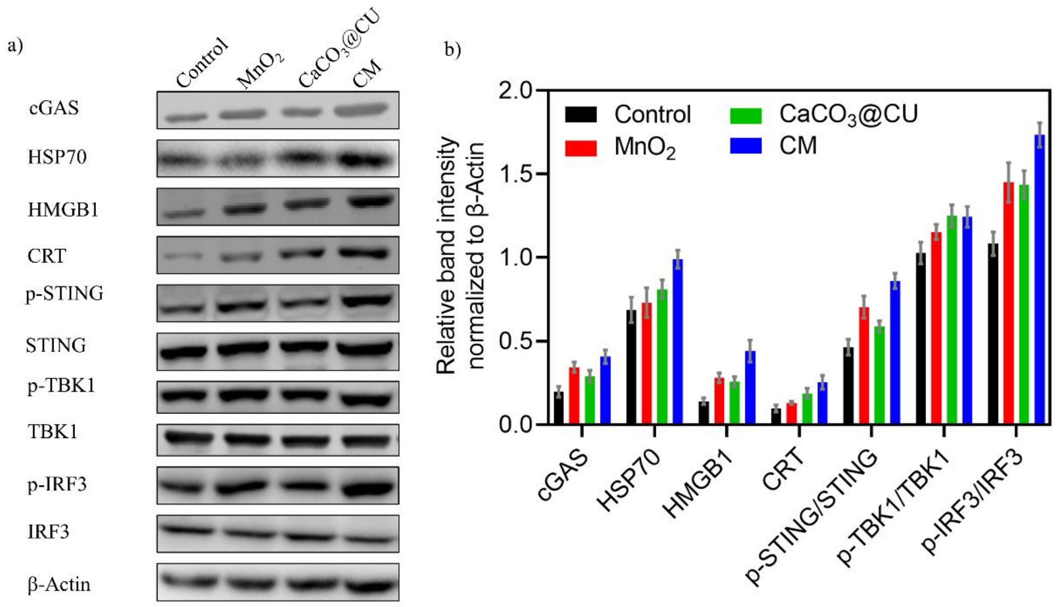

Figure S14. The western blotting results of cGAS-STING signaling pathway. a) Representative image of western blotting. b) The semi-quantification results of western blotting in S14a.

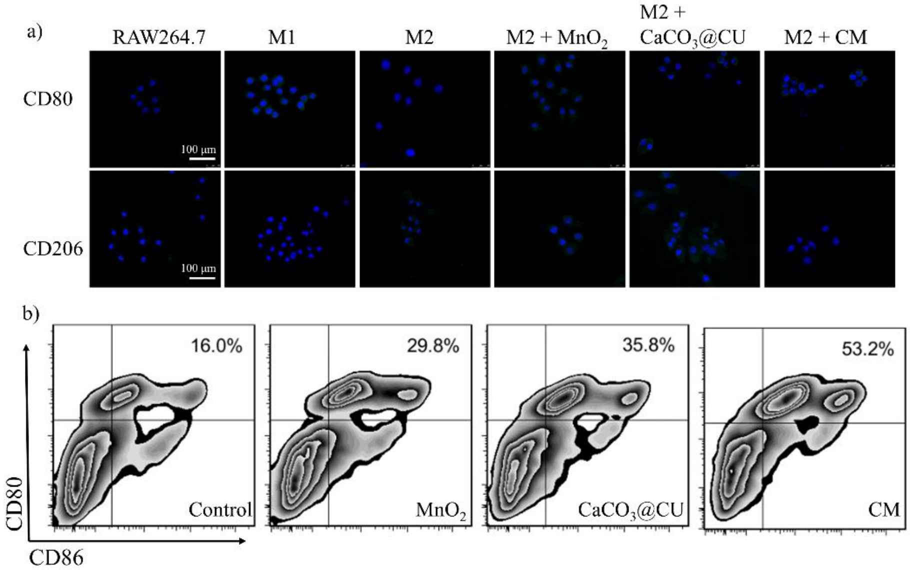

Figure S15. *In vitro* polarizing of remodeling of tumor-associated macrophages (TAMs). (a) Representative images of macrophage polarization after treated with various nanomaterials and stained with CD80 (M1) or CD206 (M2). (b) Flow cytometry analysis of M2 macrophages after B16F10 cancer cells were incubated with different nanoparticles or without treatment (M1 ($CD80^{+}CD86^{+}$) and M2 (CD206)).

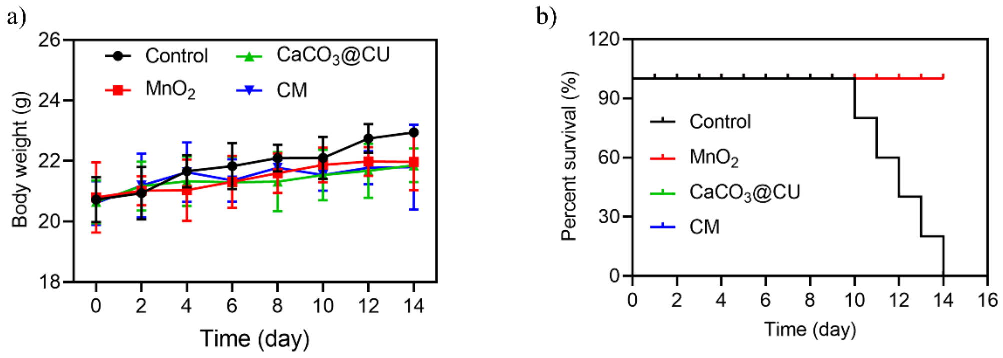

Figure S16. Body weight (a) and survival (b) change of mice in BALB/c nude mice.

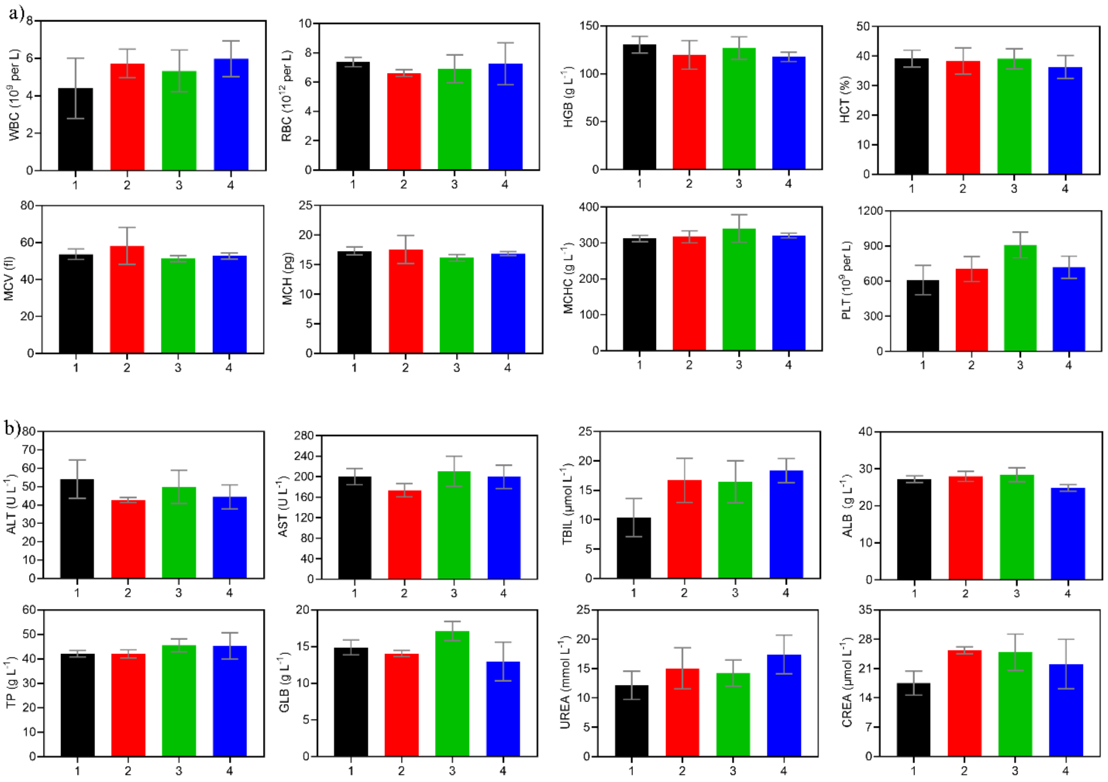

Figure S17. *In vivo* toxicology evaluation of the different treatment after 14 days injection and different treatment in BALB/c nude mice. (a) The haematological data WBC, RBC, HGB, MCV, MCHC, PLT, HCT; (b) Blood biochemical data including ALT, AST, TP, GLB, TBIL, UREA, CREA, ALB; group 1:Control, group 2:MnO_2, group 3: $CaCO_3$@CU, group 4:CM.

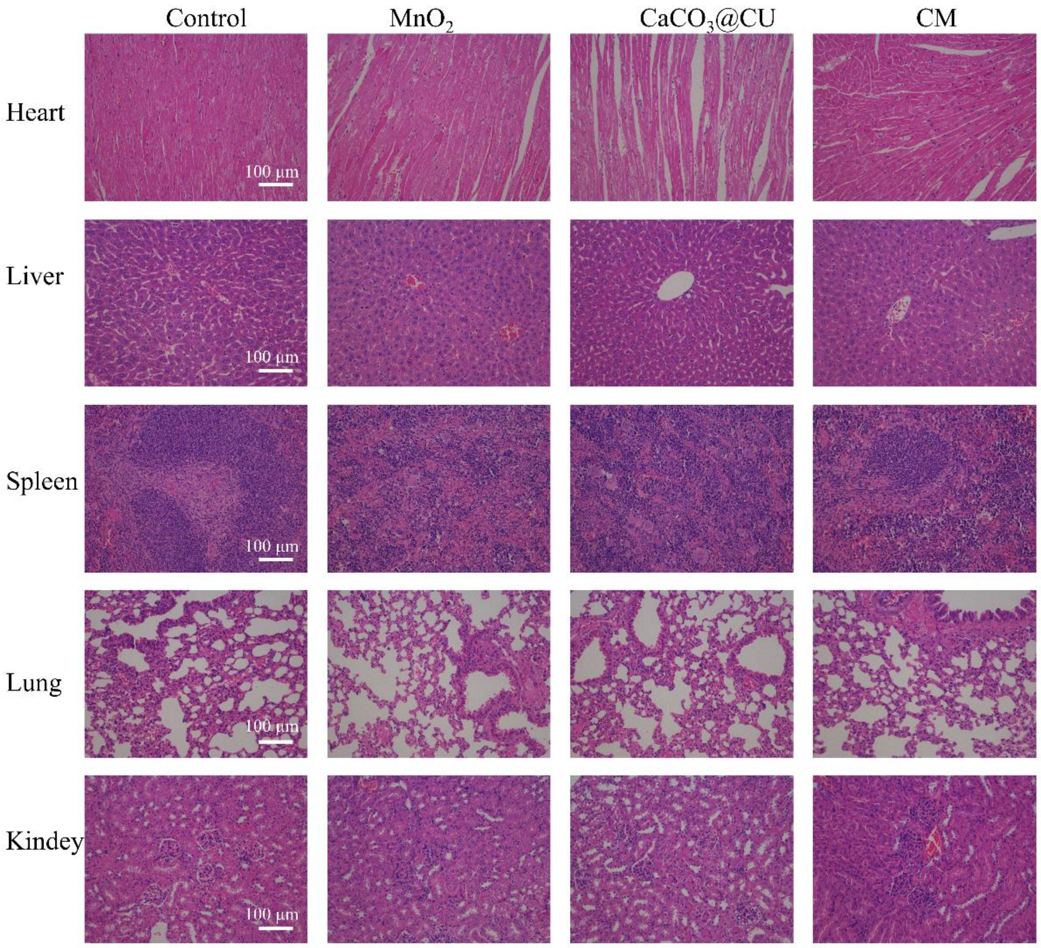

Figure S18. *In vivo* toxicology evaluation of different treatment with HE staining of the major organs including the heart, liver, spleen, lung and kidney of the mice.

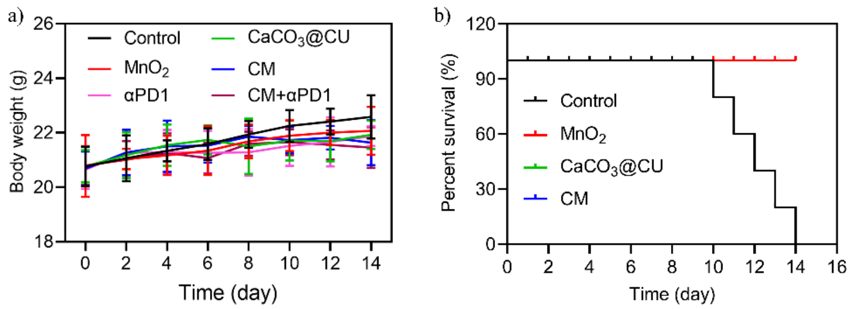

Figure S19. Body weight change (a) and survival rate (b) of B16F10 cancer cells bearing mice in C57BL/5J mice after various treatment.

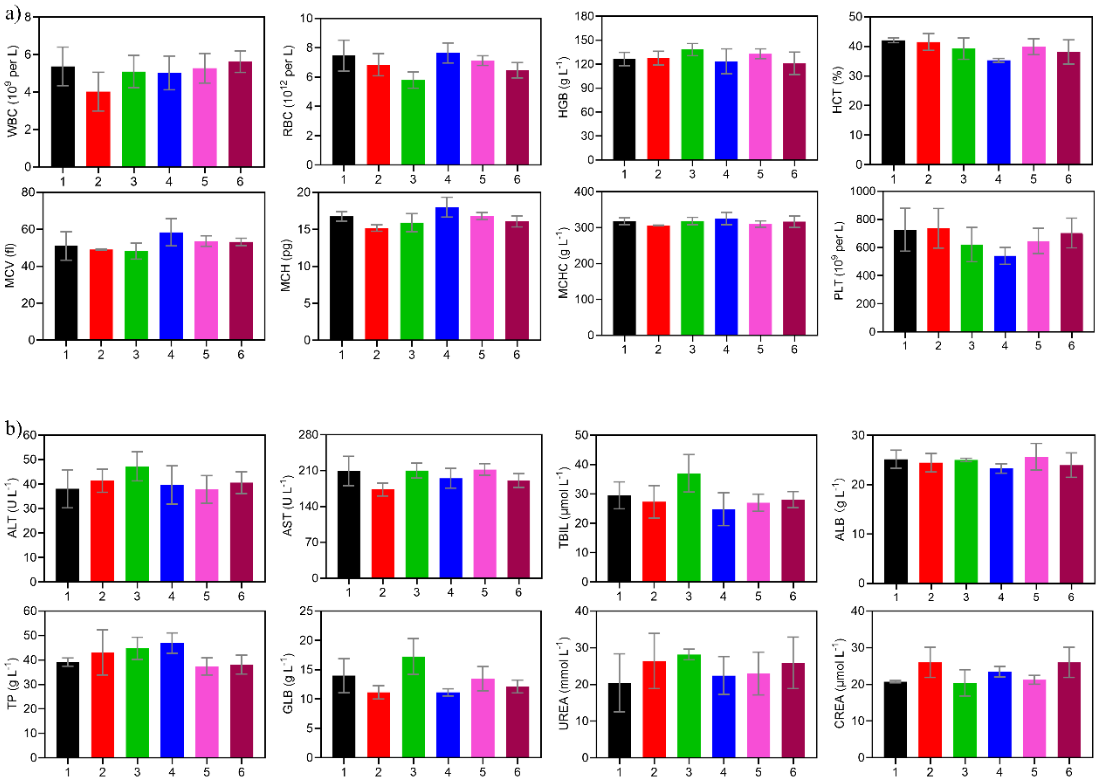

Figure S20. *In vivo* toxicology evaluation of the different treatment after 14 days injection and different treatment in B16F10 cancer cells bearing C57BL/5J mice. (a) The haematological data WBC, RBC, HGB, MCV, MCHC, PLT, HCT; (b) Blood biochemical data including ALT, AST, TP, GLB, TBIL, UREA, CREA, ALB; group 1:Control, group 2:MnO_2, group 3: $CaCO_3$@CU, group 4:CM, group 5: αPD1, group 6: CM + αPD1.

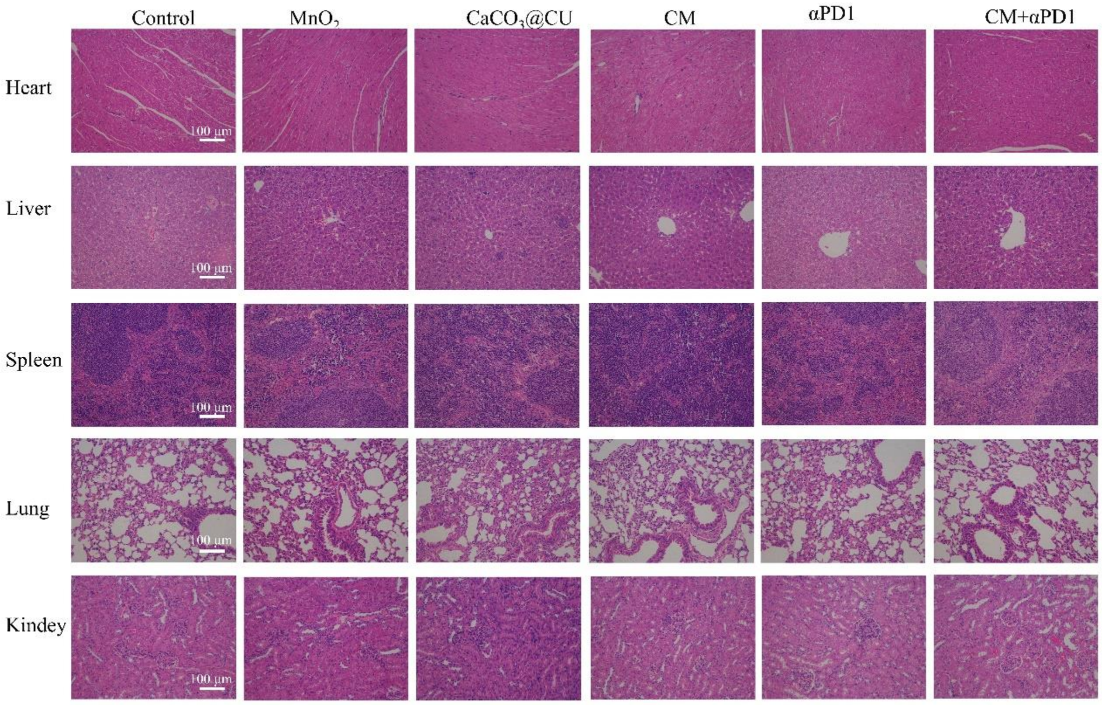

Figure S21. *In vivo* toxicology evaluation of different treatment with HE staining of the major organs including the heart, liver, spleen, lung and kidney of the mice.

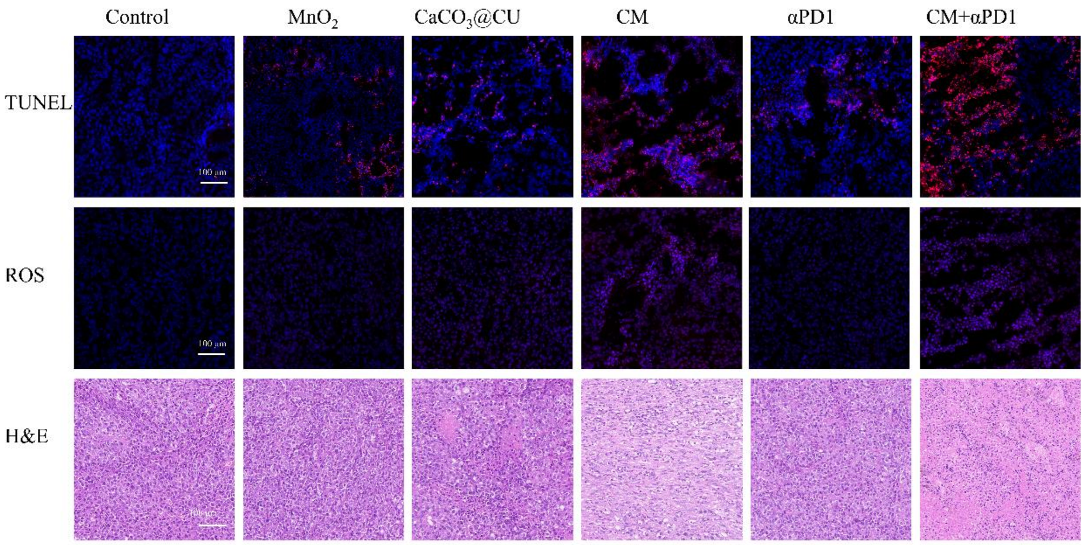

Figure S22. Tumor slice analysis. Representative images of ROS levels (red: ROS, blue: DAPI) and apoptosis based on TUNEL staining (red: apoptotic cell, blue: DAPI), and HE-stained tumor sections after different treatments.

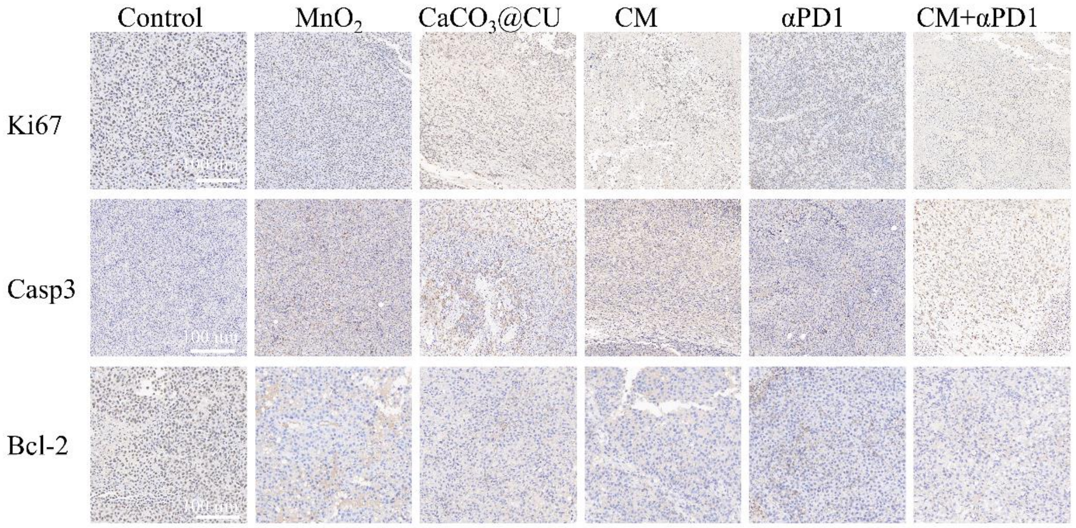

Figure S23. Representative images of cell death marker IHC staining. Ki67: proliferative protein marker; Bcl-2: antiapoptotic protein marker; Caspase-3: apoptotic protein marker.

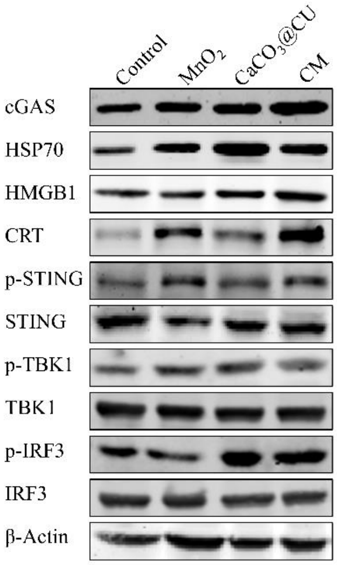

Figure S24. The western blotting results of ICD induction *in vivo*.

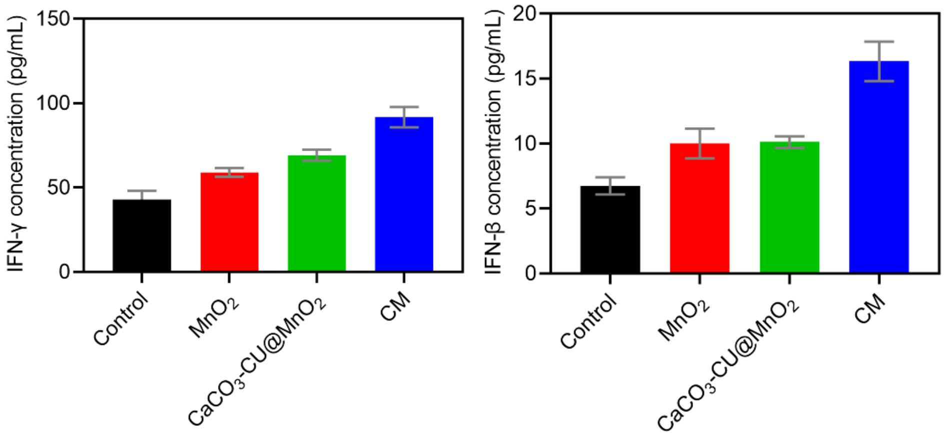

Figure S25. The in vivo examination of IFN-γ and IFN-β after different treatment.

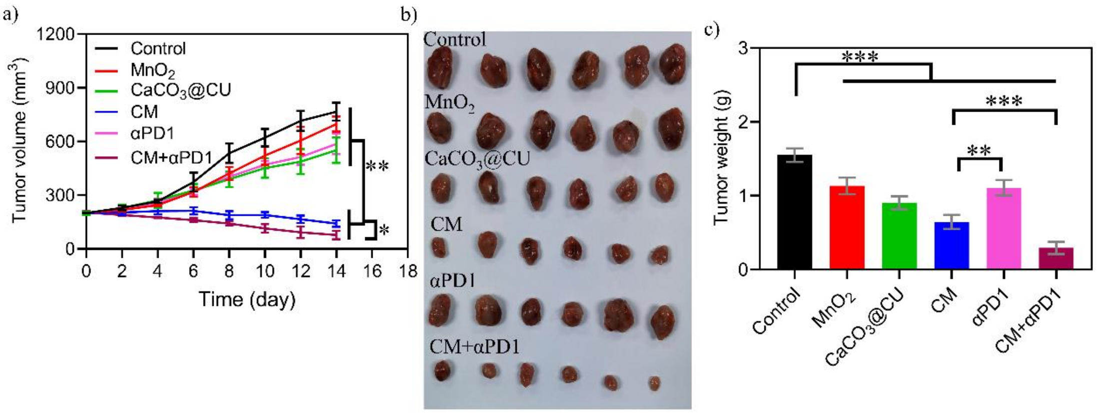

Figure S26. *In vivo* antitumor study in PANC-1 cancer cells bearing C57BL/6J mice. (a) Images of tumors obtained on day 14 post-injection; (b) Tumor volume growth curves; (c) Tumor weight analysis.

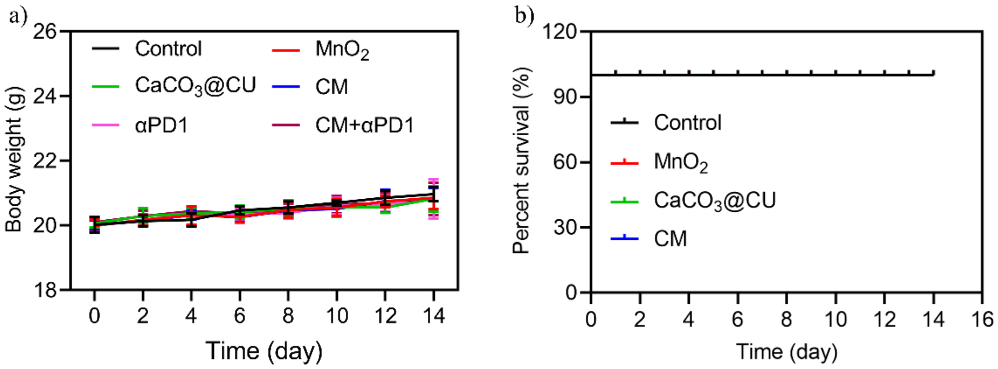

Figure S27. Body weight change (a) and survival rate (b) of PANC-1 cancer cells bearing mice in C57BL/5J mice after various treatment.

References:

(1) Ouyang, Q.; Wang, L.; Ahmad, W.; Rong, Y.; Li, H.; Hu, Y.; Chen, Q. A highly sensitive detection of carbendazim pesticide in food based on the upconversion-MnO2 luminescent resonance energy transfer biosensor. *Food chemistry* **2021**, *349*, 129157. DOI: 10.1016/j.foodchem.2021.129157.
